# Acute and Chronic Effects of Green Oat (*Avena sativa*) Extract on Cognitive Function and Mood during a Laboratory Stressor in Healthy Adults: A Randomised, Double-Blind, Placebo-Controlled Study in Healthy Humans

**DOI:** 10.3390/nu12061598

**Published:** 2020-05-29

**Authors:** David O. Kennedy, Bernd Bonnländer, Stefanie C. Lang, Ivo Pischel, Joanne Forster, Julie Khan, Philippa A. Jackson, Emma L. Wightman

**Affiliations:** 1 Brain, Performance and Nutrition Research Centre, Northumbria University, Newcastle-upon-Tyne NE1 8ST, UK; jo.forster@northumbria.ac.uk (J.F.); julie.khan@northumbria.ac.uk (J.K.); philippa.jackson@northumbria.ac.uk (P.A.J.); 2 Anklam Extrakt GmbH, Marienbergstr. 92, 90411 Nuremberg, Germany; bernd.bonnlaender@anklam-extrakt.de (B.B.); stefanie.Lang@anklam-extrakt.de (S.C.L.); 3 Research Group Pharmacognosy and Phytotherapy, UCL School of Pharmacy, London WC1N 1AX, UK; i.pischel@ucl.ac.uk; 4 NUTRAN, Northumbria University, Newcastle NE1 8ST, UK; emma.l.wightman@northumbria.ac.uk

**Keywords:** cognition, working memory, brain, stress, phytochemicals, polyphenols, triterpenes, *Avena sativa*, green oat extract

## Abstract

Green oat (*Avena sativa*) extracts contain several groups of potentially psychoactive phytochemicals. Previous research has demonstrated improvements in cognitive function following a single dose of these extracts, but not following chronic supplementation. Additionally, whilst green oat extracts contain phytochemicals that may improve mood or protect against stress, for instance species-specific triterpene saponins, to date this possibility has not been examined. The current study investigated the effects of a single dose and four weeks of administration of a novel, *Avena sativa* herbal extract (cognitaven^®^) on cognitive function and mood, and changes in psychological state during a laboratory stressor. The study adopted a dose-ranging, double-blind, randomised, parallel groups design in which 132 healthy males and females (35 to 65 years) received either 430 mg, 860 mg, 1290 mg green oat extract or placebo for 29 days. Assessments of cognitive function, mood and changes in psychological state during a laboratory stressor (Observed Multitasking Stressor) were undertaken pre-dose and at 2 h and 4 h post-dose on the first (Day 1) and last days (Day 29) of supplementation. The results showed that both a single dose of 1290 mg and, to a greater extent, supplementation for four weeks with both 430 mg and 1290 mg green oat extract resulted in significantly improved performance on a computerised version of the Corsi Blocks working memory task and a multitasking task (verbal serial subtractions and computerised tracking) in comparison to placebo. After four weeks, the highest dose also decreased the physiological response to the stressor in terms of electrodermal activity. There were no treatment-related effects on mood. These results confirm the acute cognitive effects of *Avena sativa* extracts and are the first to demonstrate that chronic supplementation can benefit cognitive function and modulate the physiological response to a stressor.

## 1. Introduction

Immature or “green” oat extracts and tinctures, made from the higher aerial parts of oat plants (*Avena sativa* L.), have a long history of medicinal use, encompassing a number of psychotropic indications, including insomnia and anxiety [1,2,3]. *Avena sativa* extracts contain a wide range of potentially bioactive secondary metabolite compounds [4] that play ecological roles for the plant [5,6,7]. These consistently include a range of terpenes, including genus-specific triterpene saponin “avenacins”, and a broad spectrum of phenolic acids and polyphenols, the latter including flavonoids and avenanthramides, a group of genus specific atypical phenolic amides [5,6,8,9,10].

These structural groups of phytochemicals include numerous compounds that have been shown to both exert wide ranging cellular and physiological effects and to modulate human brain function [7,11]. On a mechanistic level, polyphenols, including avenanthramides [8], have been shown to interact with diverse components of mammalian cellular signal transduction, including brain-specific direct and indirect interactions with neurotransmitter receptors [11,12,13,14]. Similarly, triterpenes may also modulate neurotransmission via direct receptor interactions [15,16] via inhibition of the enzymes that catalyse the oxidation or hydrolysis of neurotransmitters [7,17,18,19] or via modulation of the functioning of the glucocorticoid and estrogen systems [7,20,21], the latter due to a structural similarity to these triterpene mammalian hormones [7]. These mechanisms potentially underlie the observation of improved cognitive function following polyphenol- and triterpene-rich herbal extracts [22,23,24,25,26,27,28]. In addition, extracts of *Avena sativa*, including those from an accession of the plant material used to make the current study’s extract [29] have previously been shown to specifically inhibit the enzymes monoamine oxidase B (MAO-B) and phosphodiesterase 4 (PDE4) [30]. This adds the upregulation of monoamine neurotransmitter function to the potential mechanisms of action of this herbal extract.

Direct demonstrations of the effects of *Avena sativa* extracts include an initial study in rats that demonstrated that the lower of two doses had beneficial effects in terms of responses to stressors, aversive learning and social behaviour [31]. In humans, several placebo-controlled cross-over trials have assessed the effects of single doses of *Avena sativa* extract. In the first, an electroencephalography (EEG) study, the higher (2500 mg) of two doses of extract resulted in a pattern of modulation of cerebro-electrical activity in the frontal cortex that was interpreted as reflecting an improvement in brain function [32]. In a further study, a single dose of 1600 mg, but not a higher dose of 2400 mg *Avena sativa* extract improved the performance of a single task (Stroop) completed by 36 elderly participants with poor cognitive function [33]. Subsequently, in a more comprehensive cross-over study involving 45 middle-aged participants, researchers employed a battery of 13 computerised cognitive tasks, and found that the lower of two doses (800 mg/1600 mg) of *Avena sativa* extract increased the speed of performance across post-dose assessments on a global measure comprising speed of performance data from all of the timed tasks. The same dose was also associated with improvements on a delayed word recall task, an executive function task (Peg and Ball) and the Corsi Blocks spatial working memory task [34].

Only two studies have assessed the effects of chronic supplementation (12 weeks) with *Avena sativa* extracts. In one study, Wong et al. [35] found that 1500 mg of green oat extract resulted in improved peripheral and cerebral vasodilation as assessed by flow-mediated dilatation, and trans-cranial Doppler during hypercapnia. However, in a further study, the same administration regimen had no effect on a number of cognitive tasks assessing attention/concentration [36]. Regarding these two latter studies, it is of interest to note that the last dose of the intervention was taken a minimum of 18 h prior to the assessment, thus only allowing a measurement of the “pure” chronic effects of the treatments.

To date, there has been no demonstration of chronic cognitive benefits following green oat extracts, and no single investigation of the comparative acute, chronic and superimposed acute/chronic effects of these extracts on brain function. There have also been no human studies that have assessed the effects of green oat extracts on aspects of mood or investigated any potential protection against psychological stress similar to the effects attributed to other “adaptogenic” triterpene-containing herbal extracts.

The current dose-ranging, double-blind, placebo-controlled, parallel-groups study investigated the potential for single doses and extended daily consumption of three ascending doses (430 mg/860 mg/1290 mg) of green oat herbal extract to modulate cognitive function and attenuate the negative shift in psychological state and the physiological responses elicited by a potent novel laboratory stressor (the Observed Multitasking Stressor (OMS)). The study included an assessment of the acute (Day 1 of treatment, 2/4 h post-dose), chronic and acute/chronic superimposed (Day 29, pre-dose, and 2/4 h post-dose) effects of the three doses of green oat extract.

## 2. Methods

### 2.1. Design

The study adopted a dose-ranging, double-blind, placebo-controlled, parallel-groups design in which the acute and chronic effects of three doses of green oat extract and placebo were assessed pre-dose and at 2 h and 4 h post-dose on the first day (i.e., acute effects) and following 29 days (±3 days) consumption of the intervention.

### 2.2. Participants

A total of 132 participants, aged between 35 and 65 years who reported themselves to be in good health were randomised (placebo = 34, 430 mg = 34, 860 mg = 33, 1290 mg = 31). Participants were excluded from the study if they had any pre-existing medical condition/illness or were currently taking prescription medications which might have an impact on their ability to take part in the study, were taking any dietary supplements, had high blood pressure (systolic over 159 mm Hg or diastolic over 99 mm Hg), had a body mass index (BMI) outside of the range 18–35 kg/m^2^, were pregnant, seeking to become pregnant or lactating, had learning difficulties or dyslexia, had an uncorrected visual impairment, were smokers or regular consumers of nicotine containing products, had a history of alcohol or drug abuse, consumed caffeine in excess of 500 mg per day, had any food intolerances/sensitivities, were unable to complete all of the study assessments or were noncompliant with regards treatment consumption (<80).

**Figure 1:**
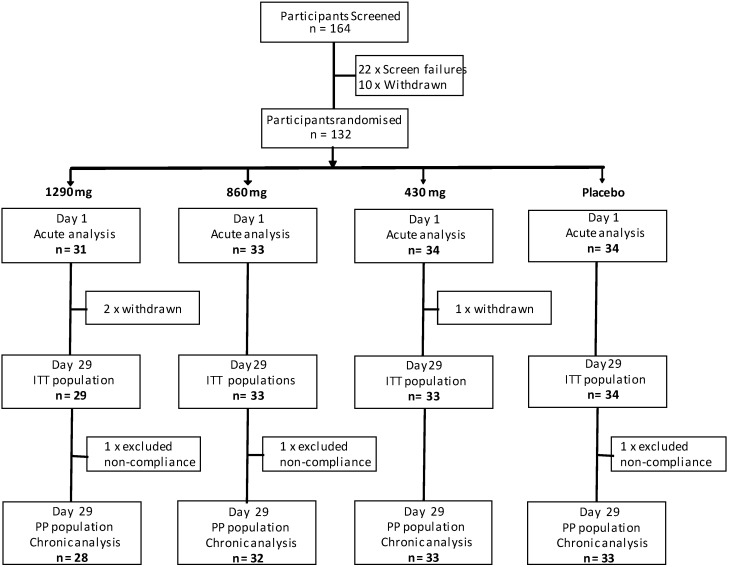
Participant dispositions throughout the trial.

All participants completed the acute (Day 1) assessment visit, with no protocol violations, and were included in the analysis of the acute effects of the treatments. A total of 129 participants went on to complete the chronic (Day 29) assessment and three participants were excluded from the chronic analysis on the basis of major protocol deviations (all compliance < 80%) leaving a Day 29 (per protocol) population of 126 participants (placebo = 33, 430 mg = 33, 860 mg = 32, 1290 mg = 28). The incidence of potential side effects (all of which were minor) did not differ significantly between treatment groups. The study dispositions are shown in Figure 1.

The demographics of the population that completed Day 1 are given in Table 1 below.

The study received ethical approval from the Northumbria University Psychology department (within the faculty of Health and Life Sciences) staff ethics committee and was conducted according to the Declaration of Helsinki (2013). All participants gave their written informed consent prior to their inclusion in the study. The trial was registered with ClinicalTrials.gov, Identifier: NCT03689348.

### 2.3. Treatments

Participants were allocated to their treatment double-blind via a computer-generated randomisation sequence.

Treatments comprised three identical, opaque light-green, hard gelatine capsules per day, with each capsule containing either placebo (maltodextrin) or 430 mg cognitaven^®^ green oat extract (Anklam Extrakt GmbH, Germany). Each green oat extract capsule contained the equivalent of 70% native *Avena sativa* extract (ethanolic (30% (m/m)): DER 4–6:1), plus excipients (maltodextrin and silicon dioxide). The capsules were manufactured under GMP-compliant conditions.

Participants were provided with three separate bottles and were instructed to consume one capsule from each bottle each morning. The combination of placebo and green oat extract capsules corresponded to a daily dose of one of the following:

- Placebo
- 430 mg cognitaven^®^ (equivalent to 300 mg native green oat extract)
- 860 mg cognitaven^®^ (600 mg native extract)
- 1290 mg cognitaven^®^ (900 mg native extract)

Participants consumed the first and last dose of their 29-day treatment regimen under supervision within the laboratory. Otherwise, they consumed their treatment at home. Compliance was checked with reference to a daily treatment diary and pill counting on the last visit to the laboratory. Tolerability and side effects were assessed with reference to standard reporting of negative health parameters. There was no significant difference in the accuracy of treatment guessing between treatments at the end of the study (see Table 1).

### 2.4. Psychological Measures

#### 2.4.1. Mood

Mood was assessed with a number of validated measures: The Bond–Lader Mood Scales [37], Profile Of Mood State (POMS) [38], State-Trait Anxiety Inventory (STAI) [39] have been described in detail elsewhere [40]). Additionally, the study utilised the General Health Questionnaire (GHQ-12) [41], an extensively used screening instrument for common mental disorders and psychiatric well-being. Each of the 12 items comprises a four-point scale (from 0 to 3) assessing the severity of a mental problem over the past few weeks. The items scores are used to generate a total score ranging from 0 to 36, with higher scores indicating poorer mental health.

#### 2.4.2. Cognitive Tasks

The cognitive tasks, with the exception of the multitasking task, were delivered using the Computerised Mental Performance Assessment System (COMPASS, Northumbria University, UK - see: www.cognitivetesting.co.uk).

The computerised tasks utilised here, in order of completion, were: Numeric Working Memory, Corsi Blocks working memory task, Rapid Visual Information Processing and the Stroop task. These cognitive tasks are described in detail elsewhere [34,42].

Additionally the study employed the following novel multitasking task.

#### 2.4.3. Multitasking Task

This multitasking task comprised the concomitant performance of two tasks at the same time in three concurrent blocks of four minutes.

Serial subtractions: During each four-minute block, participants were instructed to count out loud backwards in 3 s, 7 s, or 17 s from a given randomly generated number between 800 and 999, as quickly and accurately as possible. Prior to commencing the task, they were instructed verbally that if they made a mistake they should carry on subtracting from the new incorrect number. The order in which participants completed the three variants of the task (3 s/7 s/17 s) was counterbalanced across participants. Performance of the task was recorded and scored for the total number of subtractions and the number of incorrect subtractions.

Tracking: Whilst performing the three blocks of verbal Serial Subtraction tasks, participants also completed a computerised tracking task, which required the participants to use the mouse to move a cursor to track an asterisk as it moved across the screen. On screen, the asterisk moved at a rate of approximately 6 cm/s on a 35 cm laptop screen (~168 pixels/s) in a smooth random path. Participants were instructed to keep the cursor as close to the asterisk as possible. The distance between the target and the cursor was computed every 100 ms and the resulting data converted to an accuracy score representing the distance of the cursor from the asterisk in pixels, averaged across the four-minute block of task performance.

As the serial subtraction and tracking tasks were always performed concomitantly, any treatment related effects could take the form of a modulation of either one or both tasks, in either direction, including the potential for a trade-off between performance of the two tasks (e.g., improvements in one task at the expense of decrements in the other task). Accuracy (errors) and speed (number of subtractions) for the subtraction tasks, and tracking accuracy (distance from target) during each subtraction task were therefore converted to standardised *Z* scores (with a higher score always representing improved performance) to allow the data from both tasks to be analysed together in one analysis. As either speed or accuracy could vary for the subtraction tasks, two separate analyses were then carried out on the acute and chronic data sets: accuracy from both tasks combined, and subtraction speed and tracking accuracy combined.

#### 2.4.4. Observed Multi-Tasking Stressor (OMS)

Interview-style laboratory stressors, such as the classic Trier Social Stress Test [43], rely on an element of surprise (prior to free speech and mental arithmetic) and are not typically repeated more than once. Computerised multitasking stressors, in which participants perform multiple on-screen tasks at once, engender very mild stress responses, but these responses can be sustained across multiple applications (e.g., [40,44,45,46]). The OMS combines elements of both of these laboratory stressors, and comprises an extended period of multitasking (verbal serial subtractions plus a concomitant computerised tracking task) whilst being observed by a panel of three researchers and video recorded in a mock interview situation. Pilot data shows that this stressor consistently provokes both psychological and physiological stress responses across multiple applications on multiple days, making it ideal for measuring stress responses across time both in an acute and chronic context (see also: Supplementary Online Materials, Section I).

The chair of the observation panel timed the tasks and provided verbal instructions (starting number, number to subtract) to the participant. All panel members made occasional notes. The computer screen, showing the tracking task, was projected onto a wall-mounted screen to give the impression that the panel was closely monitoring the participant’s performance.

The effect of the stressor on the psychological state of participants was assessed by the completion of the STAI and Bond–Lader mood scales immediately before and immediately after completion. In terms of physiological stress responses, galvanic skin response (GSR) and heart rate (HR) were measured throughout the OMS via a monitor on the index finger of the participant’s nondominant hand (Vilistus Digital Sampling Unit, Durham Systems Management Ltd., Penrith, UK). Saliva samples were collected using Salivettes (Sarstedt Ltd., Leicester, UK) before and after the stressor, and analysed using ELISA (Salimetrics Ltd., Newmarket, UK) for cortisol and α-amylase levels using standard methodology.

### 2.5. Procedure

Cognitive testing took place in a suite of testing facilities with participants visually isolated from each other. OMS testing took place in an interview room. Participants attended the laboratory on three separate occasions: an introductory visit between 1 and 14 days before the first day of treatment, and two testing days (Day 1 and Day 29 with respect to treatment commencing).

The introductory visit to the laboratory comprised briefing on the requirements of the study, obtaining of informed consent, health screening, completion of the Caffeine Consumption Questionnaire, and training on the cognitive and mood measures and collection of demographic data.

**Figure 2:**
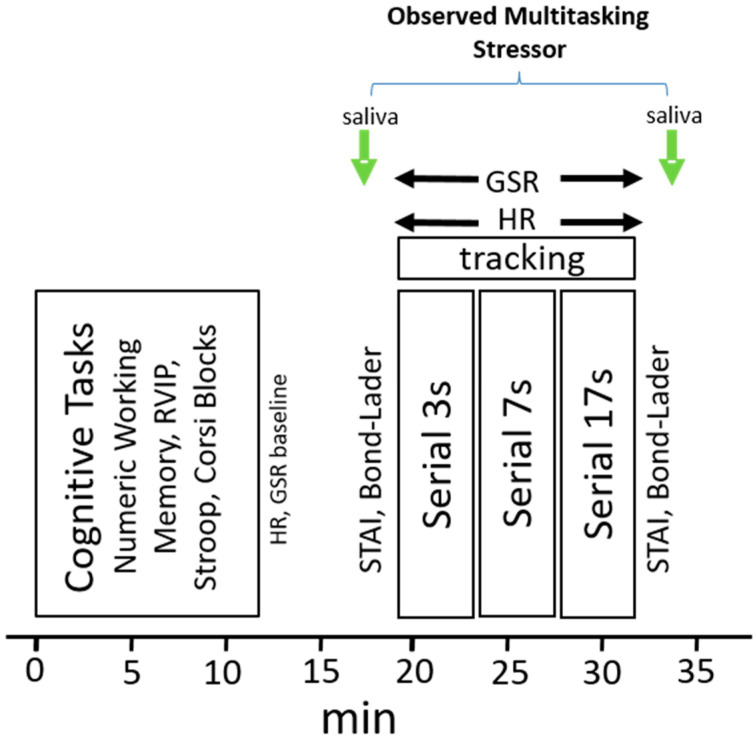
Cognitive and Observed Multitasking Stressor (OMS) assessment. Participants completed a cognitive assessment (Numeric Working memory, Stroop, RVIP, Corsi Blocks) in the general laboratory and then moved to the interview room. They provided a saliva sample (cortisol/α-amylase) and then completed the STAI-state and Bond–Lader mood scales before and after the completion of three four-minute verbal serial subtraction tasks (serial 3 s, 7 s, 17 s, completed in counterbalanced order) and a concomitant computerised tracking task. Heart rate (HR) and galvanic skin response (GSR) were measured throughout performance of the tasks.

For the two subsequent laboratory-based testing sessions (Day 1, Day 29) participants attended the laboratory before 8.00 a.m., having consumed a standard breakfast of cereal and/or toast at home no later than an hour before arrival. They were required to have refrained from alcohol for 24 h and caffeine for 18 h prior to attendance. The procedure during the Day 1 and Day 29 visits was identical. On arrival on each day, participants completed the GHQ-12 and POMS mood measures. Each subsequent assessment comprised the COMPASS cognitive tasks (Corsi Blocks, Stroop, RVIP, Numeric Working Memory—14 min) followed by collection of resting baseline heart rate and galvanic skin response data. Participants then moved to the interview room and completed the Observed Multitasking Stressor (OMS). The 15-min OMS took place in front of a panel of three observers, as described above, and comprised provision of a saliva sample and completion of STAI-state and Bond–Lader mood scales that were completed before and after the stressor. The stressor comprised the performance of three concurrent separate blocks of verbal serial subtraction tasks (Serial 3 s, Serial 7 s, Serial 17s–4 min per block) whilst concomitantly performing a computerised tracking task. Heart rate and galvanic skin response (GSR) were recorded throughout. Figure 2 depicts the running order of assessment.

**Figure 3:**
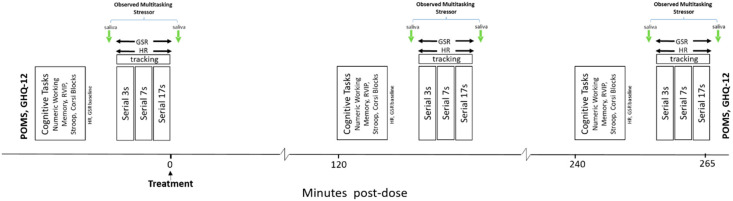
Schedule of each testing session (Day 1, Day 29). Participants completed the POMS and GHQ-12, followed by the pre-dose cognitive/OMS assessment. After this, they took their day’s treatment, and then completed further identical cognitive/OMS assessments commencing 2 h and 4 h post-dose. Before departing, they completed the POMS and GHQ-12.

After the first cognitive/OMS assessment, participants took their treatment for the day and then underwent identical cognitive/OMS assessments commencing 2 h and 4 h post-dose. Following completion of the final assessment, participants completed the GHQ-12 and POMS (to assess any general effects on anxiety, depression, and mood). Figure 3 shows the timeline of the Day 1 and Day 29 testing days.

### 2.6. Analysis

Prior to the primary analysis of the effects of treatment, pre-dose baseline (Day 1 pre-dose assessment) differences between treatment were investigated by one-way (treatment (0, 430, 860, 1290 mg)) or two way (pre/post-OMS × treatment) ANOVA.

Given that the POMS and GHQ-12 were simply completed before treatment and at the end of testing on each assessment day this data was analysed with a two-factor (pre/post-treatment × Day 1/29) ANOVA.

For all other measures, two analyses were conducted: the ACUTE analysis interrogated Day 1 data (2 h and 4 h post-dose assessments, using pre-dose baseline data as a covariate) and the CHRONIC analysis interrogated Day 29 data (pre-dose, 2 h and 4 h post-dose, using Day 1 pre-dose baseline data as a covariate). The primary approach to both analyses was via analysis of covariance. For those measures with a single baseline covariate that applied to all levels of all repeated measures factors (i.e., COMPASS cognitive tasks), the analysis was by General Linear Model (GLM) ANCOVA with Day 1 pre-dose baseline data as covariate. For those measures that had multiple baseline covariates that applied to one of the repeated measures factors (Multitasking, GSR/HR/cortisol/amylase, change in mood during stressor), analysis was by Linear Mixed Models (LMM; compound symmetry) using the MIXED procedure in SPSS (version 22.0, IBM corp., Armonk, NY, USA). For the measures with pre and post stressor measures (cortisol/amylase, change in mood during stressor), terms were fitted for ‘treatment’ (0, 430, 860, 1290 mg), ‘assessment’ (ACUTE Day 1 analysis—2 h/4 h post-dose: CHRONIC Day 29 analysis—pre-dose, 2 h/4 h post-dose), ’pre/post OMS‘ and their two and three way interactions. For the multitasking, GSR and h the ’pre/post OMS‘ factor was replaced with a ’task‘ (serial 3 s/7 s/17 s) factor, and multitasking had an additional ’outcome‘ (subtractions/tracking) factor. In all analyses, given the potential for most of the outcomes to vary with age, the participant’s age was entered as a second covariate.

For both ACUTE and CHRONIC analyses, predefined planned comparisons (*t*-tests calculated with the pooled variance) between placebo and each dose of green oat extract were conducted in order to answer two key questions: (1) Did the green oat extract treatments differ from placebo when data was averaged across the assessments included in the analyses (i.e., exploring the main effect of treatment); (2) Did the green oat extract treatments differ from placebo during each of the assessments included in the analysis. In the case of the dual-task data, which comprised standardised (*Z* score) data from two concomitant tasks, additional planned comparisons were conducted that parsed the contributions of each task.

Only those planned comparisons associated with a measure that evinced a treatment related main effect or interaction effect on the initial analysis are reported here. Cohen’s d (d) effect sizes were calculated for each significant planned comparison.

## 3. Results

### 3.1. Baseline Differences

There were no significant baseline differences on any measure.

### 3.2. Cognitive Function

#### 3.2.1. Corsi Blocks Working Memory Task

**Figure 4:**
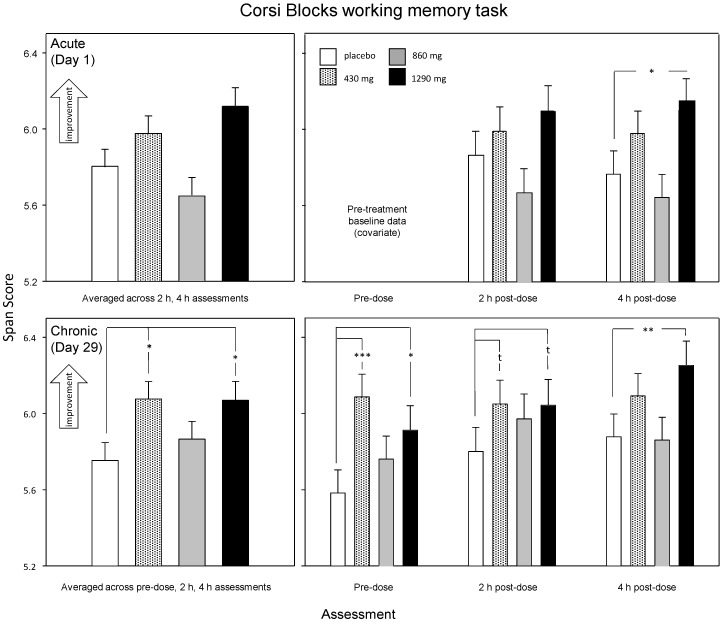
Corsi Blocks working memory task. The effects of treatment from the acute (Day 1) analysis (top panels) and the chronic (Day 29) analysis. Data are estimated means (plus SE) derived from the ANCOVA/LMM analysis, using pretreatment (Day 1 pre-dose assessment) data and participant’s age as covariates. The left-hand panels show the results of planned comparisons between placebo and each dose of green oat extract using data averaged across the day, and the right-hand panels shows planned comparisons during each individual assessment. t, *p* < 0.1; *, *p* < 0.05; **, *p* < 0.01; ***, *p* < 0.001 in comparison to placebo.

Acute (Day 1) analysis: There was a significant main effect of treatment [F (3, 123) = 2.82, *p* = 0.042) on the Corsi Blocks working memory task span score. Reference to the planned comparisons of data from the individual assessments showed that 1290 mg green oat extract resulted in improved working memory (span score) during the 4 h post-dose assessment (*p* = 0.014: *d* = 0.4) only (see Figure 4). There were no significant differences between placebo and any of the active treatments when span scores were averaged across assessments.

Chronic (Day 29) analysis: The initial ANCOVA demonstrated a significant main effect of treatment (F (3, 119) = 2.85, *p* = 0.04) on the Corsi Blocks span score. Planned comparisons showed that following both the 430 mg and 1290 mg doses of green oat extract, participants’ scores were increased overall (430 mg, *p* = 0.017: *d* = 0.64; 1290 mg *p* = 0.02: *d* = 0.61). With reference to the individual assessments, 430 mg green oat extract resulted in improved span during the pre-dose assessment (*p* < 0.001) (d = 0.79) with a trend towards the same effect during the 2 h post-dose assessment (*p* = 0.062: *d* = 0.35), whilst 1290 mg resulted in an improved score during the pre-dose (*p* = 0.015: *d* = 0.47) and 4 h post-dose (*p* = 0.005: *d* = 0.53) assessments with a trend (*p* = 0.07) (*d* = 0.32) towards the same effect at the 2 h post-dose assessment (see Figure 4).

See Online Supplementary Tables S1 and S2 for cognitive task data.

#### 3.2.2. Multitasking Task

**Figure 5:**
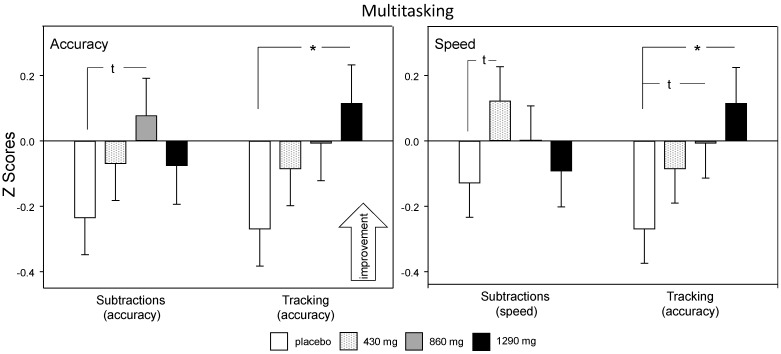
Acute (Day 1) treatment related change in multitasking performance averaged across 2 h and 4 h post-dose assessments. Left panel: subtractions accuracy (errors) and tracking accuracy, Right panel: subtraction speed (total number) and tracking accuracy. Scores for each measure were converted into standardised *Z* score, with a positive score indicating improved performance in order to analyse data together. Data shown are estimated means (plus SE) derived from the LMM analysis, using pretreatment baseline data and age as covariates. The data for tracking represented in both panels is the same, with differences in the planned comparisons relating to differences in variance across both tasks. t, *p* < 0.1; *, *p* < 0.05 in comparison to placebo at that time point.

Acute (Day 1) analysis: There was a significant treatment × outcome interaction with respect to accuracy on the subtraction (errors) and tracking (distance from target) tasks (F (3, 1404.6) = 2.640, *p* = 0.048). Reference to the planned comparisons showed that whilst 1290 mg green oat extract did not result in significantly increased overall accuracy, it did result in improved tracking accuracy (*p* = 0.018: *d* = 0.59). There was also a strong trend towards significantly increased subtraction task accuracy following 860 mg green oat extract (*p* = 0.051: *d* = 0.48). It is noteworthy that subtraction accuracy was numerically higher than placebo in all of the green oat extract treatment groups in comparison to placebo, excluding the possibility that tracking was improved at the expense of subtractions (see Figure 5).

As the subtraction speed/tracking accuracy analysis used the same tracking data, there was a similar treatment × outcome interaction (F (3, 1404.6) = 8.264, *p* < 0.001). The pattern here was largely the same with significant improvements only seen on the tracking task following 1290 mg green oat extract (*p* = 0.011: *d* = 0.63) with a trend towards the same effect following 860 mg (*p* = 0.078: *d* = 0.4). Regarding the speed of subtraction performance, only 430 mg evinced a trend towards significance (*p* = 0.078: *d* = 0.42), but all active treatments were numerically superior to placebo, confirming that speed of subtraction performance was not compromised at the expense of tracking accuracy (see Figure 5).

See Online Supplementary Tables S3 and S4 for Day 1 multitasking data.

**Figure 6:**
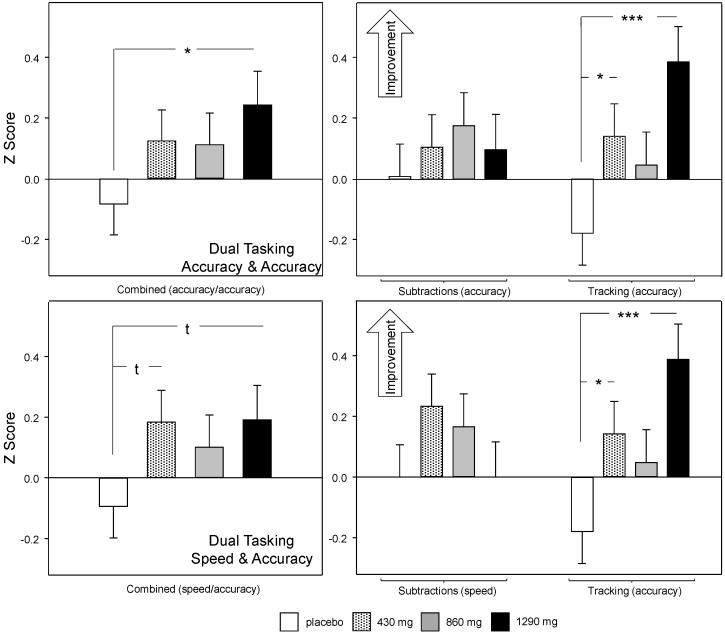
Chronic (Day 29) treatment related change in multitasking performance averaged across pre-dose, 2 h and 4 h post-dose assessments. Scores for each measure were converted into standardised *Z* score with a positive score indicating improved performance in order to analyse data together. Data shown are estimated means (plus SE) derived from the LMM analysis, using Day 1 pretreatment baseline data and age as covariates. The *Z* scores are plotted with an increased score indicating improved performance. The top panels show subtraction accuracy (errors) and tracking accuracy data; the bottom panels show subtraction speed (total number) and tracking accuracy data. In both top and bottom panels the left-hand panels show comparisons of mean Day 29 data averaged across the two concomitantly performed task outcomes (serial subtractions/tracking), and the right-hand panel shows comparisons conducted on the separate task outcomes. The data for tracking is the same in both analyses. t, *p* < 0.1, *, *p* < 0.05; ***, *p* < 0.001 in comparison to placebo.

Chronic (Day 29) analysis: The initial analysis showed that there was a significant treatment × outcome interaction in terms of accuracy on the two tasks (F (3, 2067.12) = 10.5, *p* < 0.001). The planned comparisons of data averaged across the two measures (top-left panel of Figure 6) showed that 1290 mg green oat extract resulted in increased overall accuracy (*p* = 0.03: *d* = 0.58). Reference to comparisons conducted on data from the individual outcomes (Figure 6, top-right panel) showed that this effect was predominantly due to improved tracking task performance following 1290 mg green oat extract (*p* < 0.001: *d* = 0.94) with an additional significant improvement on this measure following 430 mg (*p* = 0.032: *d* = 0.54). Of note, given that subtraction accuracy was numerically higher in all the green oat extract treatments than in placebo, the possibility that improved tracking had a detrimental influence on subtraction performance can be excluded.

A similar interaction (F (3, 2067.12) = 20.406, *p* < 0.001) was also evident in the analysis of subtraction speed (total number of subtractions) and tracking accuracy. Here, there was only a trend towards improved performance in data averaged across the two tasks for 430 mg (*p* = 0.06: *d* = 0.49) and 1290 mg (*p* = 0.06: *d* = 0.48), but a comparable pattern as described above for the accuracy/accuracy analysis, with significant improvements only seen on the tracking task (1290 mg (*p* < 0.001: *d* = 0.63); 430 mg (*p* = 0.03: d = 0.55)). Again, this data confirms that speed of subtraction performance was not compromised at the expense of tracking.

**Figure 7:**
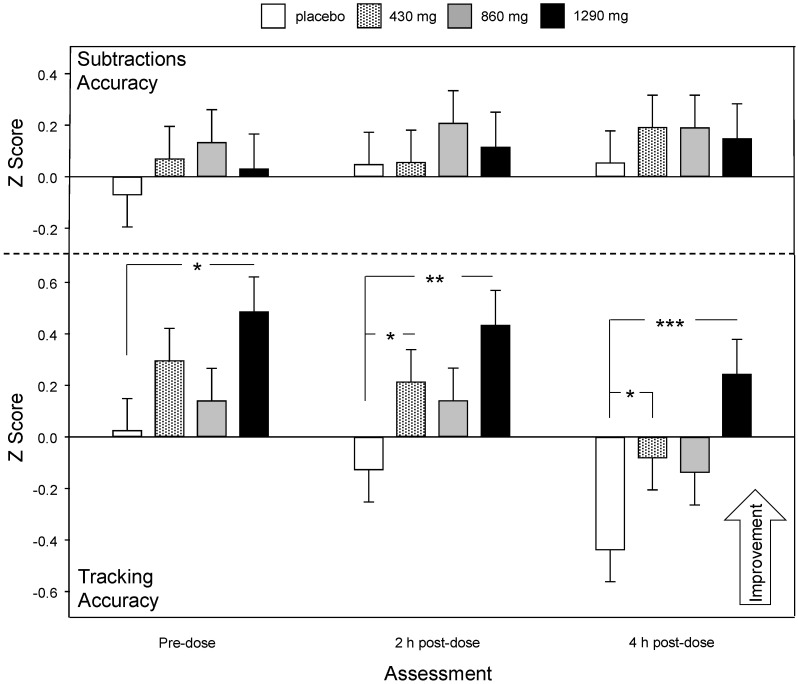
Chronic (Day 29) treatment related change in multitasking performance (accuracy of subtractions and tracking) during the OMS pre-dose and during the 2 h and 4 h post-dose assessments. Data are estimated means (plus SE) of *Z* score data, derived from the LMM analysis, using pretreatment (Day 1 pre-dose assessment) data and participant’s age as covariates. The *Z* scores are plotted with an increased score indicating improved performance. The top panels show subtraction accuracy (errors) the bottom panels show tracking accuracy data from a single LMM analysis. t, *p* < 0.1, *, *p* < 0.05; **, *p* < 0.01; ***, *p* < 0.001 in comparison to placebo.

There was also an interaction between treatment, outcome and assessment (F (8, 2066.418) = 4.63, *p* < 0.001), suggesting that the modulation of the effect of treatment on tracking accuracy changed as a function of time (i.e., assessment). Reference to planned comparisons of group means for the tracking data (from the subtraction accuracy/tracking accuracy analysis) between the groups during each assessment showed that the benefits following 1290 mg green oat extract were evident pre-dose (*p* = 0.012: *d* = 0.65) and became stronger (after taking the day’s treatment) during the 2 h post-dose (*p* = 0.002: *d* = 0.79) and 4 h post-dose assessments (*p* < 0.001: *d* = 0.96). Whilst consuming 430 mg did not result in any improvement pre-dose, it did lead to an improvement during the 2 h post-dose (*p* = 0.05: *d*) = 0.49 and 4 h post-dose (*p* = 0.041: *d* = 0.5) assessments (see Figure 7).

The subtraction speed/tracking accuracy analysis incorporated the same tracking data and returned the same pattern of results (not reported for brevity). See Online Supplementary Tables S5 and S6 for Day 29 multitasking data.

#### 3.2.3. Other Cognitive Tasks

There were no other significant effects on any of the other cognitive task outcomes, with the exception of a significant treatment × assessment interaction (F (6, 232) = 2.2, *p* = 0.044) with regards the % accuracy of RVIP task performance on Day 29. However, reference to the data showed that this effect related solely to a seemingly anomalous reduction in performance following 860 mg (*p* < 0.001, *d* = 0.62) during the 4 h post-dose assessment. There were no other significant differences on this measure, or any other indications of performance decrements across the cognitive tasks that supported this finding.

### 3.3. Mood

The Observed Multitasking Stressor had the expected effect on mood, leading to increased anxiety as assessed by the STAI-state and decreased ratings of “Contentedness” or “Calmness” as assessed by the Bond–Lader mood scales during each assessment on Day 1 and Day 29 (see Supplementary Online materials Section I for details). However, there was no treatment related modulation of the changes in mood as a result of the OMS stressor, or in resting mood (POMS and GHQ-12). See Online Supplementary Tables S7 (OMS Day 1), S8 (OMS Day 29), S9 (POMS) and S10 (GHQ-12) for data.

### 3.4. Physiological Measures

The only acute (Day 1) physiological effect was a significant treatment × assessment interaction with regards heart rate (change from resting baseline) during the OMS stressor on Day 1 (F (3, 632.759) = 4.465, *p* = 0.004). Whilst there were no significant differences between treatments averaged across assessments, a single treatment (860 mg green oat extract) was associated with an increase in heart rate (*p* = 0.036: *d* = 0.52), in comparison to placebo during the 4 h post-dose assessment.

**Figure 8:**
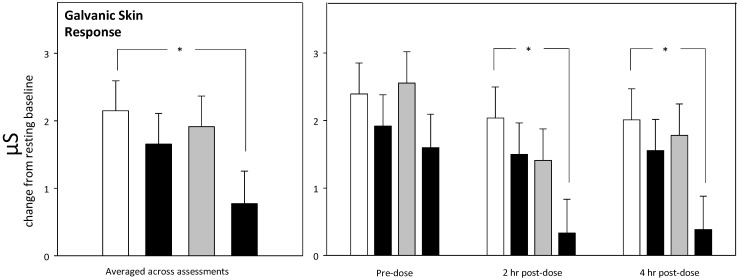
Chronic (Day 29) treatment-related change in the electrodermal galvanic skin response to the stressor. Data are change from resting baseline estimated means (plus SE) derived from the LMM analysis, using pretreatment (Day 1 pre-dose assessment) data and age as covariates. The left-hand panel shows planned comparisons between group means averaged across the testing day, and the right panel shows planned comparisons during each individual assessment. *, *p* < 0.05 in comparison to placebo.

With regards the chronic (Day 29) analysis, there was a treatment × assessment interaction (F (6, 1000) = 3.578, *p* = 0.002) in terms of the galvanic skin response during the OMS stressor. Reference to the planned comparisons of data averaged across the assessments showed that 1290 mg green oat extract was associated with a decreased electrodermal response to the stressor in comparison to placebo (*p* = 0.038: *d* = 0.54) across assessments. When looking at individual assessments, these effects were evident during both the 2 h (*p* = 0.013: *d* = 0.64) and 4 h post-dose (*p* = 0.017: *d* = 0.62) assessments (see Figure 8).

There was no chronic treatment related effect on heart rate, and no acute or chronic effect on cortisol or α-amylase responses to the stressor.

See Online Supplementary Tables S11/S12 (GSR/HR) and S13/S14 (cortisol and α-amylase) for data.

## 4. Discussion

In the current study, both a single dose and 29 days of supplementation with *Avena sativa* green oat extract resulted in dose-dependent improvements in cognitive function. The beneficial effects were strongest following the highest dose of the extract (1290 mg), but were also evident following the lowest (430 mg) dose. Regarding longer term (29 days) supplementation, cognitive benefits were seen both before taking the day’s dose of the intervention and thereafter they increased in strength during the post-dose assessments (2 h post-dose and 4 h post-dose). Whilst the OMS laboratory stressor had the expected anxiogenic effects across the study assessments, green oat extract did not attenuate this shift in mood, or have any effects on mood in the absence of the stressor. However, after consuming the highest dose of the extract for 29 days, the participants’ electrodermal stress response during the stressor was attenuated.

Looking in more detail at the cognitive improvements, benefits were seen both in terms of performance of a computerised version of the Corsi Blocks spatial working memory task and in tracking performance during extended multitasking. The highest dose (1290 mg) of green oat extract resulted in some acute benefits (Corsi Blocks and tracking) on Day 1. However, following 29 days of supplementation, enhanced performance of both tasks was seen following both 430 mg and 1290 mg of the extract before taking the day’s treatment, suggesting a “pure chronic” effect independent of any acute effect of consuming the treatment. Thereafter, the pattern of effects after four weeks for the highest dose, and to some extent the lower dose, was observed to be markedly stronger at 2 h and 4 h post-dose. Given the pre-dose effects and the pattern of enhanced benefits after four weeks, it seems reasonable to conclude that the extract resulted in chronic cognitive benefits, which were augmented either by long-term administration, or by the superimposition of additional acute benefits on Day 29.

Interestingly, the cognitive benefits seen here were dose-dependent, but did not follow a linear dose–response curve. Indeed the effects were seen following the lowest and highest dose, but were not evident for the middle dose (860 mg) of green oat extract. Whilst this may simply be due to the margin of error in measuring human cognitive function, it is also worth noting that U-shaped dose responses (i.e., effects at low and high, but not interim doses) are commonly observed in biological systems [47]. Inverse, nonlinear, bimodal and U-shaped dose responses have also been described in animals and humans following diverse phytochemicals [22,26,48,49], including *Avena sativa* extracts [33,34]. The same has been seen with regards potential modulatory mechanisms. For instance, herbal extracts such as *Ginkgo biloba* have been shown to induce Cytochrome p450 enzymes in a biphasic and nonlinear manner [50].

Previous research has shown that single doses of *Avena sativa* extract can result in improved cognitive function (the Stroop task) in cognitively compromised elderly participants [33], with broader cognitive benefits seen in healthy middle-aged (40 to 65 years) participants [34]. However, whilst chronic administration of *Avena sativa* extract for 12 weeks increased peripheral and cerebral vasodilation [35], the same administration regimen had no effect on cognitive performance [36]. The current study is therefore the first to demonstrate a beneficial effect of chronic supplementation with *Avena sativa* extract on cognitive function.

Interestingly, the most striking benefits seen here following green oat extract supplementation were in terms of dual-tasking performance, which may be seen as providing a more ecologically valid example of real-world cognitive demands than single cognitive tasks. The verbal/computerised dual-task paradigm used here could be conceived as mirroring the cognitive demands of many everyday situations. As an example, driving performance is typically degraded by engaging in conversation (i.e., a secondary verbal task) within the car or on the phone [51]. It is notable that in the current study, improved tracking was not at the cost of subtraction performance, which was numerically improved for all three doses of the extract. Clearly, the ability to multitask more effectively could be conceived as a concrete benefit of this green oat extract applicable to many real-world situations. Evidence suggests that the ability to multitask is best predicted by working memory performance [52]. In the current study green oat extract also resulted in improved working memory task performance and these two findings may be related.

In terms of mood, there were no significant effects of treatment on any variable. The current study adopted a novel methodology that allowed the investigation of the potential attenuation by the extract of the psychological and physiological consequences of completing a laboratory-based stressor. The OMS proved to be effective as a psychological stressor across visits and assessments (increased anxiety and decreased calmness and contentedness; see Online Supplementary Materials), but green oat extract (1290 mg) only resulted in an attenuation of the GSR electrodermal skin conductance response to the stressor. This electrodermal response, related as it is to sweat gland activity, is regarded as a good biomarker for sympathetic nervous system activation [53] and is a reliable indicator of increased stress, arousal, emotion and even anxiety [53,54,55]. However, the latter finding is somewhat difficult to interpret in the absence of any attenuation of the psychological consequences of the stressor. However, the overall pattern of results does not exclude the possibility that this extract might have beneficial mood effects (in line with the somewhat limited animal data to date [31]), in those suffering from poor mood, high anxiety, or affective disorders. Clearly this possibility requires further investigation.

Given the complex phytochemistry of *Avena sativa* extracts, it is difficult to elucidate the exact mechanisms of action underpinning the cognitive benefits seen here. The extract’s ability to inhibit MAO-B, an enzyme that metabolises dopamine, which itself plays a key role in working memory and executive function [56,57], is certainly a candidate as the primary mechanism here. The inhibition of the enzyme PDE4, which hydrolyses the cellular second messenger cyclic adenosine monophosphate (cAMP), might be expected to principally affect long-term memory [58], which was not measured in this study. However, the mechanisms of action of the various classes of phytochemicals typically found in *Avena sativa* extracts may also apply here. As an example, polyphenols, a class of phytochemicals, which would include the avenanthramides and flavonols/flavones found abundantly in *Avena sativa* extracts, owe their bioactivity to interaction with components of a range of cellular signal transduction pathways. In the brain, the net effect of these interactions include the increased synthesis of brain growth factors, such as neurotrophins and the vasodilatory molecule nitric oxide, which play a pivotal role in cerebral blood flow regulation [11,12,13,14], and direct interactions within the cellular signalling cascades triggered by receptor interactions, giving these molecules the potential to modulate diverse aspects of neuronal function [11]. These processes may underlie the observations in the literature of both acute [22] and chronic [23,24] modulation of cognitive function by polyphenol-rich plant extracts. Triterpenes, such as the avenacins found in *Avena sativa* extracts, also have the potential to modulate brain function via diverse mechanisms, including direct and indirect modulation of glucocorticoid and estrogenic function [7,20,21], modulation of neurotransmission via direct receptor interactions [15,16] and inhibition of the enzymes such as MAO-B and AChE, which catalyse the oxidation or hydrolysis of numerous neurotransmitters [7,17,18,19]. Again, mechanisms such as these may account for the improved cognitive function seen following triterpene-rich herbal extracts [25,26,27,28], and may also account for the effects seen here following green oat extract.

Naturally, the study had several limitations that deserve discussion. The interpretation of the results is complicated by the lack of a straightforward linear dose response. However, it is notable that the clearest results were seen following the highest dose of extract (1290 mg), and it would seem reasonable to conclude that the maximum effect lies at or above this dose. In any study employing multiple measures of mood/psychological state and batteries of cognitive tasks (in this case, five tasks), it also is not possible to entirely rule out the possibility of interactions in ratings/performance related to completing similar measures. However, there is no reason to think that such a theoretical possibility might interact with the effects of an intervention. This is also the first publication to report multiple applications of the novel OMS stressor, and whilst the psychological effects of this stressor were as expected, we await demonstrations that these acute anxiogenic effects are amenable to attenuation by a nutritional intervention.

In conclusion, single doses and four weeks of supplementation with green oat extract resulted in significant benefits to cognitive function, both in terms of working memory and dual-task (working memory/executive function and tracking) performance. These beneficial cognitive effects are broadly in line with previous demonstrations of improved cognitive task performance following single doses of other *Avena sativa* extracts. These findings also represent the first demonstration of significant cognitive benefits following chronic (in this case four weeks) supplementation. The benefits to multitasking performance, which may represent a more ecologically valid measure of everyday cognitive function than traditional “single task” cognitive assessments, is of particular interest, and deserves further research attention.

## Figures and Tables

**Table 1:** Demographic details of the randomised participants (i.e., Day 1—acute analysis sample). There were no significant differences between groups on any measure. Note: the doses of finished product correspond to doses of 300, 600 and 900 mg native extract respectively).

	Treatment Group			
	Placebo	430 mg	860 mg	1290 mg
Age at Enrolment (years)	49.39	49.35	49.52	47.21
Gender	F25/M9	F21/M13	F23/M10	F23/M8
Years in Education	16.84	16.88	17.14	15.28
Portions of Fruit & Veg	4.23	4.15	4.59	4.32
Alcohol consumption daily (units)	0.99	0.99	0.66	0.83
Caffeine Consumption (mg/day)	207	194	219	243
Blood Pressure—Systolic	122	122	119	124
Blood Pressure—Diastolic	80	81	78	80
Heart Rate (bpm)	72	75	72	69
Body Mass Index	26.43	26.87	25.45	25.93
Treatment guess (% placebo)	47%	52%	36%	38%

## Supplementary Materials

The following are available online at https://www.mdpi.com/2072-6643/12/6/1598/s1. Section I: Observed Multitasking Stressor (OMS), Table S1: Acute (Day 1) effects of green oat extract on cognitive task performance, Table S2: Chronic (Day 29) effects of green oat extract on cognitive task performance, Table S3: Acute (Day 1) effects of green oat extract on multitasking accuracy (subtractions and tracking), Table S4: Acute (Day 1) effects of green oat extract on multitasking speed (number of subtractions) and accuracy (tracking),Table S5: Chronic (Day 29) effects of green oat extract on multitasking accuracy (subtractions and tracking), Table S6: Chronic (Day 29) effects of green oat extract on multitasking speed (subtractions) and accuracy (tracking), Table S7: Acute (Day 1) effects of green oat extract on the change in mood during the OMS, Table S8: Chronic (Day 29) effects of green oat extract on the change in mood during the OMS, Table S9: Profile of Mood States (POMS) sub-factor scores, Table S10: General Health Questionnaire-12 (GHQ-12) scores (PP population),Table S11: Acute (Day 1) effects of green oat extract on galvanic skin response and heart data during the stressor, Table S12: Chronic (Day 29) effects of green oat extract on galvanic skin response and heart data, Table S13: Acute (Day 1) effects of green oat extract on salivary cortisol and α-amylase, Table S14: Chronic (Day 29) effects of green oat extract on salivary cortisol and α-amylase.

## References

[1] Abascal K., Yarnell E. (2004). Nervine herbs for treating anxiety. Altern. Complementary Ther..

[2] Blumenthal M., Goldberg A., Brinckmann J. (2000). Herbal Medicine. Expanded Commission e Monographs.

[3] Kaur D., Kamboj A., Shri R. (2016). Comparative evaluation of anxiolytic effects of various extracts of oats (avena sativa), rice bran (oryza sativa) and spinach (spinacia oleracea) in experimental animals. Int. J. Pharm. Sci. Res..

[4] Osbourn A.E. (2003). Saponins in cereals. Phytochemistry.

[5] Meydani M. (2009). Potential health benefits of avenanthramides of oats. Nutr. Rev..

[6] Mylona P., Owatworakit A., Papadopoulou K., Jenner H., Qin B., Findlay K., Hill L., Qi X., Bakht S., Melton R. (2008). Sad3 and sad4 are required for saponin biosynthesis and root development in oat. Plant Cell.

[7] Kennedy D.O. (2014). Plants and the Human Brain.

[8] Sur R., Nigam A., Grote D., Liebel F., Southall M.D. (2008). Avenanthramides, polyphenols from oats, exhibit anti-inflammatory and anti-itch activity. Arch. Dermatol. Res..

[9] Bahraminejad S., Asenstorfer R., Riley I., Schultz C. (2008). Analysis of the antimicrobial activity of flavonoids and saponins isolated from the shoots of oats (avena sativa l.). J. Phytopathol..

[10] Günther-Jordanland K., Dawid C., Dietz M., Hofmann T. (2016). Key phytochemicals contributing to the bitter off-taste of oat (avena sativa l.). J. Agric. Food Chem..

[11] Kennedy D.O. (2014). Polyphenols and the human brain: Plant “secondary metabolite” ecologic roles and endogenous signaling functions drive benefits. Adv. Nutr..

[12] Spencer J.P. (2009). Flavonoids and brain health: Multiple effects underpinned by common mechanisms. Genes Nutr..

[13] Williams R.J., Spencer J.P. (2012). Flavonoids, cognition, and dementia: Actions, mechanisms, and potential therapeutic utility for alzheimer disease. Free Radic. Biol. Med..

[14] Baptista F.I., Henriques A.G., Silva A.M., Wiltfang J., Da Cruz e Silva O.A. (2014). Flavonoids as therapeutic compounds targeting key proteins involved in alzheimer’ s disease. ACS Chem. Neurosci..

[15] Ramasamy S., Chin S.P., Sukumaran S.D., Buckle M.J.C., Kiew L.V., Chung L.Y. (2015). In silico and in vitro analysis of bacoside a aglycones and its derivatives as the constituents responsible for the cognitive effects of bacopa monnieri. PLoS ONE.

[16] Ryoo N., Rahman M.A., Hwang H., Ko S.K., Nah S.-Y., Kim H.-C., Rhim H. (2019). Ginsenoside rk1 is a novel inhibitor of nmda receptors in cultured rat hippocampal neurons. J. Ginseng Res..

[17] Armijos C., Gilardoni G., Amay L., Lozano A., Bracco F., Ramirez J., Bec N., Larroque C., Finzi P.V., Vidari G. (2016). Phytochemical and ethnomedicinal study of huperzia species used in the traditional medicine of saraguros in southern ecuador; ache and mao inhibitory activity. J. Ethnopharmacol..

[18] Bahadori M.B., Dinparast L., Valizadeh H., Farimani M.M., Ebrahimi S.N. (2016). Bioactive constituents from roots of salvia syriaca l.: Acetylcholinesterase inhibitory activity and molecular docking studies. S. Afr. J. Bot..

[19] Singh R., Ramakrishna R., Bhateria M., Bhatta R.S. (2014). In vitro evaluation of bacopa monniera extract and individual constituents on human recombinant monoamine oxidase enzymes. Phytother. Res..

[20] Francis G., Kerem Z., Makkar H.P., Becker K. (2002). The biological action of saponins in animal systems: A review. Br. J. Nutr..

[21] Gao X.-Q., Du Z.-R., Yuan L.-J., Zhang W.-D., Chen L., Teng J.-J., Wong M.-S., Xie J.-X., Chen W.-F. (2019). Ginsenoside rg1 exerts anti-inflammatory effects via g protein-coupled estrogen receptor in lipopolysaccharide-induced microglia activation. Front. Neurosci..

[22] Scholey A.B., French S.J., Morris P.J., Kennedy D.O., Milne A.L., Haskell C.F. (2010). Consumption of cocoa flavanols results in acute improvements in mood and cognitive performance during sustained mental effort. J. Psychopharmacol..

[23] Mastroiacovo D., Kwik-Uribe C., Grassi D., Necozione S., Raffaele A., Pistacchio L., Righetti R., Bocale R., Lechiara M.C., Marini C. (2014). Cocoa flavanol consumption improves cognitive function, blood pressure control, and metabolic profile in elderly subjects: The cocoa, cognition, and aging (cocoa) study—A randomized controlled trial. Am. J. Clin. Nutr..

[24] Desideri G., Kwik-Uribe C., Grassi D., Necozione S., Ghiadoni L., Mastroiacovo D., Raffaele A., Ferri L., Bocale R., Lechiara M.C. (2012). Benefits in cognitive function, blood pressure, and insulin resistance through cocoa flavanol consumption in elderly subjects with mild cognitive impairmentnovelty and significance the cocoa, cognition, and aging (cocoa) study. Hypertension.

[25] Kongkeaw C., Dilokthornsakul P., Thanarangsarit P., Limpeanchob N., Norman Scholfield C. (2014). Meta-analysis of randomized controlled trials on cognitive effects of bacopa monnieri extract. J. Ethnopharmacol..

[26] Reay J.L., Kennedy D.O., Scholey A.B. (2005). Single doses of panax ginseng (g115) reduce blood glucose levels and improve cognitive performance during sustained mental activity. J. Psychopharmacol..

[27] Reay J.L., Kennedy D.O., Scholey A.B. (2006). Effects of panax ginseng, consumed with and without glucose, on blood glucose levels and cognitive performance during sustained ‘mentally demanding’ tasks. J. Psychopharmacol..

[28] Ossoukhova A., Owen L., Savage K., Meyer M., Ibarra A., Roller M., Pipingas A., Wesnes K., Scholey A. (2015). Improved working memory performance following administration of a single dose of american ginseng (panax quinquefolius l.) to healthy middle-age adults. Hum. Psychopharmacol. Clin. Exp..

[29] Lang S.C. (2017). Avena sativa: A natural supporter of cognition and mental fitness. Wellness Foods & Supplements.

[30] Moccetti T., Wullschleger C., Schmidt A., Aydogan C., Kreuter M. (2006). Bioactivity-based development of a wild green oat (avena sativa l.) extract in support of mental health disorders. Z. Für Phytother..

[31] Schellekens C., Perrinjaquet-Moccetti T., Wullschleger C., Heyne A. (2009). An extract from wild green oat improves rat behaviour. Phytother. Res..

[32] Dimpfel W., Storni C., Verbruggen M. (2011). Ingested oat herb extract (avena sativa) changes eeg spectral frequencies in healthy subjects. J. Altern. Complementary Med..

[33] Berry N.M., Robinson M.J., Bryan J., Buckley J.D., Murphy K.J., Howe P.R. (2011). Acute effects of an avena sativa herb extract on responses to the stroop color–word test. J. Altern. Complementary Med..

[34] Kennedy D.O., Jackson P.A., Forster J., Khan J., Grothe T., Perrinjaquet-Moccetti T., Haskell-Ramsay C.F. (2017). Acute effects of a wild green-oat (avena sativa) extract on cognitive function in middle-aged adults: A double-blind, placebo-controlled, within-subjects trial. Nutr. Neurosci..

[35] Wong R.H., Howe P.R., Coates A.M., Buckley J.D., Berry N.M. (2013). Chronic consumption of a wild green oat extract (neuravena) improves brachial flow-mediated dilatation and cerebrovascular responsiveness in older adults. J. Hypertens..

[36] Wong R.H., Howe P.R., Bryan J., Coates A.M., Buckley J.D., Berry N.M. (2012). Chronic effects of a wild green oat extract supplementation on cognitive performance in older adults: A randomised, double-blind, placebo-controlled, crossover trial. Nutrients.

[37] Bond A., Lader M. (1974). Use of analog scales in rating subjective feelings. Br. J. Med Psychol..

[38] McNair P.M., Lorr M., Droppleman L. (1992). Poms Manual: Profile of Mood States.

[39] Spielberger C., Gorsuch R., Lushene R. (1969). The State Trait Anxiety Inventory Manual.

[40] Haskell C.F., Robertson B., Jones E., Forster J., Jones R., Wilde A., Maggini S., Kennedy D.O. (2010). Effects of a multi-vitamin/mineral supplement on cognitive function and fatigue during extended multi-tasking. Hum. Psychopharmacol..

[41] Goldberg D.P., Williams P. (1988). A User’s Guide to the General Health Questionnaire.

[42] Kennedy D., Wightman E., Khan J., Grothe T., Jackson P. (2019). The acute and chronic cognitive and cerebral blood-flow effects of nepalese pepper (zanthoxylum armatum dc.) extract—A randomized, double-blind, placebo-controlled study in healthy humans. Nutrients.

[43] Kirschbaum C., Pirke K.-M., Hellhammer D.H. (1993). The ‘trier social stress test’—A tool for investigating psychobiological stress responses in a laboratory setting. Neuropsychobiology.

[44] Scholey A., Haskell C., Robertson B., Kennedy D., Milne A., Wetherell M. (2009). Chewing gum alleviates negative mood and reduces cortisol during acute laboratory psychological stress. Physiol. Behav..

[45] Kennedy D.O., Little W., Haskell C.F., Scholey A.B. (2006). Anxiolytic effects of a combination of melissa officinalis and valeriana officinalis during laboratory induced stress. Phytother. Res..

[46] Kennedy D.O., Little W., Scholey A.B. (2004). Attenuation of laboratory-induced stress in humans after acute administration of melissa officinalis (lemon balm). Psychosom. Med..

[47] Calabrese E.J., Baldwin L.A. (2001). U-shaped dose-responses in biology, toxicology, and public health. Annu. Rev. Public Health.

[48] Kay C.D., Hooper L., Kroon P.A., Rimm E.B., Cassidy A. (2012). Relative impact of flavonoid composition, dose and structure on vascular function: A systematic review of randomised controlled trials of flavonoid-rich food products. Mol. Nutr. Food Res..

[49] Fiebich B.L., Knörle R., Appel K., Kammler T., Weiss G. (2011). Pharmacological studies in an herbal drug combination of st. John’s wort (hypericum perforatum) and passion flower (passiflora incarnata): In vitro and in vivo evidence of synergy between hypericum and passiflora in antidepressant pharmacological models. Fitoterapia.

[50] Hellum B.H., Hu Z., Nilsen O.G. (2007). The induction of cyp1a2, cyp2d6 and cyp3a4 by six trade herbal products in cultured primary human hepatocytes. Basic Clin. Pharmacol. Toxicol..

[51] Caird J.K., Simmons S.M., Wiley K., Johnston K.A., Horrey W.J. (2018). Does talking on a cell phone, with a passenger, or dialing affect driving performance? An updated systematic review and meta-analysis of experimental studies. Hum. Factors.

[52] Bühner M., König C.J., Pick M., Krumm S. (2006). Working memory dimensions as differential predictors of the speed and error aspect of multitasking performance. Hum. Perform..

[53] Boucsein W. (2012). Electrodermal Activit.

[54] Christopoulos G.I., Uy M.A., Yap W.J. (2019). The body and the brain: Measuring skin conductance responses to understand the emotional experience. Organ. Res. Methods.

[55] Kyriakou K., Resch B., Sagl G., Petutschnig A., Werner C., Niederseer D., Liedlgruber M., Wilhelm F.H., Osborne T., Pykett J. (2019). Detecting moments of stress from measurements of wearable physiological sensors. Sensors.

[56] Arnsten A.F. (1998). Catecholamine modulation of prefrontal cortical cognitive function. Trends Cogn. Sci..

[57] Robbins T.W., Arnsten A.F. (2009). The neuropsychopharmacology of fronto-executive function: Monoaminergic modulation. Annu. Rev. Neurosci..

[58] Blokland A., Van Duinen M.A., Sambeth A., Heckman P.R., Tsai M., Lahu G., Uz T., Prickaerts J. (2019). Acute treatment with the pde4 inhibitor roflumilast improves verbal word memory in healthy old individuals: A double-blind placebo-controlled study. Neurobiol. Aging.

