# Supplementary material for: Acute and Chronic Effects of Green Oat (Avena sativa) Extract on Cognitive Function and Mood during a Laboratory Stressor in Healthy Adults: A Randomised, Double-Blind, Placebo-Controlled Study in Healthy Humans
Source: Nutrients. 2020 May 29;12(6):1598. doi: 10.3390/nu12061598 (PMC7352613; doi:10.3390/nu12061598)
Supplement: Supplementary file 1 [file nutrients-12-01598-s001.pdf]

nutrients

MDPI

Acute and chronic effects of *Avena sativa* extract on cognitive function and mood during a laboratory stressor in healthy adults: a randomized, double-blind, placebo-controlled study in healthy humans

ONLINE SUPPLEMENTARY MATERIALS

Section I

Observed Multi-tasking Stressor (OMS)

'Interview' style laboratory stressors, such as the classic Trier Social Stress Test [1], rely on an element of surprise (prior to free speech and mental arithmetic) and are not typically repeated over time. Computerised multi-tasking stressors, in which participants perform multiple on-screen tasks at once, engender very mild stress responses, but these responses can be sustained across multiple applications (e.g. [2-5]). The OMS combines elements of both of these laboratory stressors, and comprises an extended period of multi-tasking (verbal Serial Subtractions plus a concomitant computerised tracking task) whilst being observed by a panel of three researchers and video recorded in a mock interview situation. The effects of the stressor on psychological state (State-Trait Anxiety Inventory and Bond-Lader Mood Scales – measured immediately before and after the stressor) on both days of the study (Day 1/Day 29) and the three assessments per day (pre-dose, 2 hr post-dose, 4 hr post-dose) are shown in Figures S1, S2, S3, S4 below. Given that there were no treatment related effects on these outcomes, the analysis reported below was by three-way [day (Day1/Day29) x 'assessment' (pre-dose/ 2hr/4hr post-dose) x 'pre/post OMS'] repeated measures ANOVA (GLM - IBM SPSS version 22.0, IBM corp.).

The results show that undergoing the Observed Multi-tasking Stressor had the expected effects on the psychological state of the participants. With reference to a three-way ANOVA of data from before and after the OMS during the three assessments on each of the two visits, completion of the OMS lead to a consistent increase in anxiety (STAI), and decrease in ratings of calmness and contentedness (Bond-Lader) . In the case of the latter two measures this effect had attenuated to a certain extent by the last assessment on Visit 2. Alertness (Bond-Lader) was also reduced by performing the OMS. However, reference to the full data shows that this effect was only apparent during the first two assessments of each visit, possibly due to its superimposition on the natural decrease in alertness seen during the testing visits. Data from the STAI and Bond-Lader Mood Scales is presented below, along with the statistics from the ANOVAs.

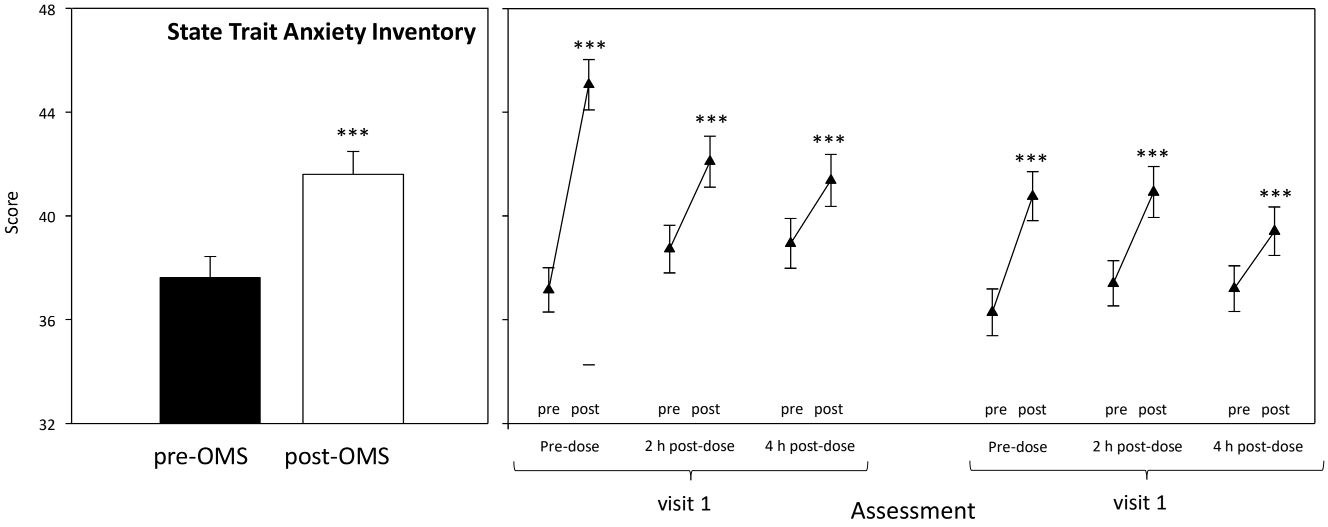

Figure 1. The effects of completing the OMS stressor on anxiety. Left panel: the main effect of OMS completion averaged across all assessments [F (1, 250) = 161.8, p < 0.001]. Right panel: the pre/post x assessment x visit interaction [F (2, 250) = 11.4, p < 0.001], showing ratings of anxiety before and after the OMS during each assessment on both visits. Asterisks denote a significant difference between pre-OMS and post-OMS ratings during that assessment. ***, p < 0.001.

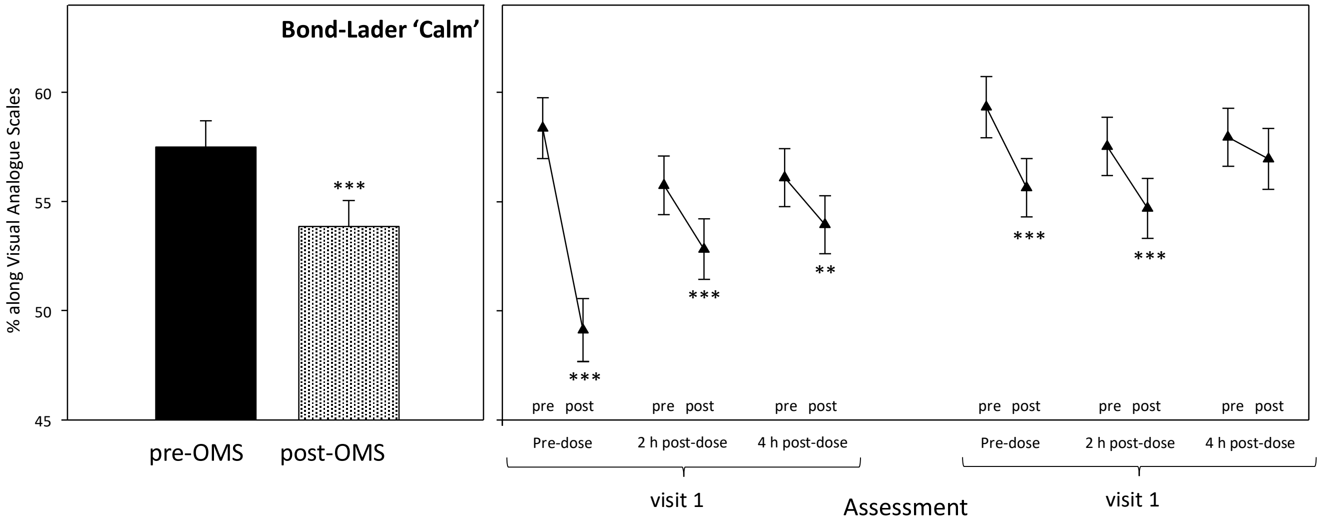

Figure 2. The effects of completing the OMS stressor on ratings of calmness (Bond-Lader Mood Scales). Left panel: the main effect of OMS completion averaged across all assessments [F (1, 246) = 57.4, p < 0.001]. Right panel: the pre/post x assessment x visit interaction [F (2, 246) = 7.8, p < 0.001], showing ratings of calmness before and after the OMS during each assessment on both visits. Asterisks denote a significant difference between pre-OMS and post-OMS ratings during that assessment. **, p < 0.01; ***, p < 0.001.

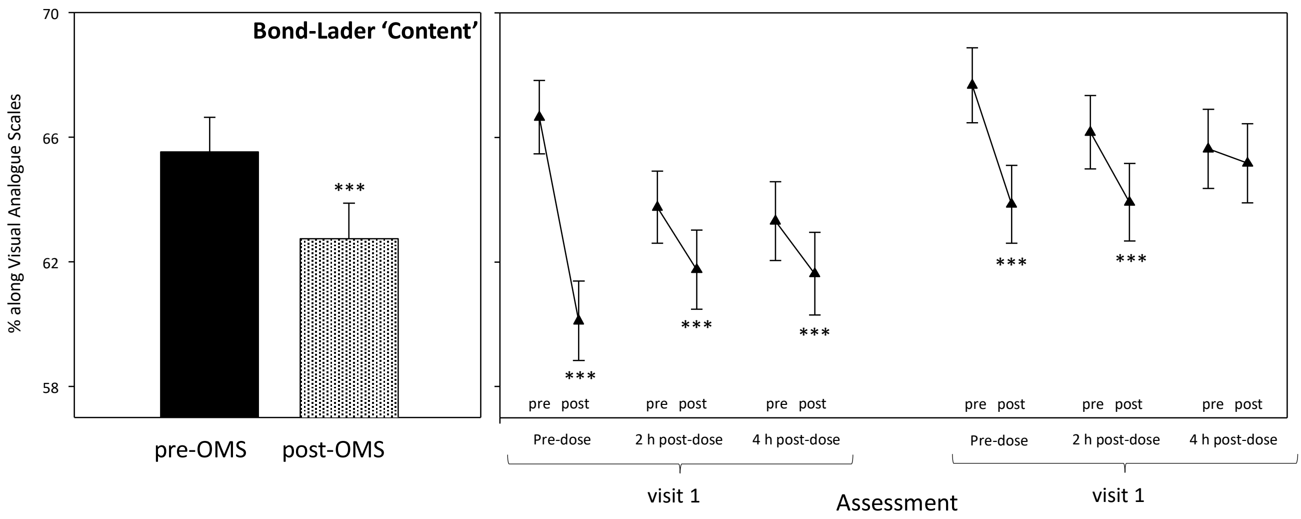

Figure 3. The effects of completing the OMS stressor on ratings of contentedness (Bond-Lader Mood Scales). Left panel: the main effect of OMS completion averaged across all assessments [F (1, 246) = 67.3, p < 0.001]. Right panel: the pre/post x assessment x visit interaction [F (2, 246) = 4.6, p =0.012], showing ratings of calmness before and after the OMS during each assessment on both visits. Asterisks denote a significant difference between pre-OMS and post-OMS ratings during that assessment. **, p < 0.01; ***, p < 0.001.

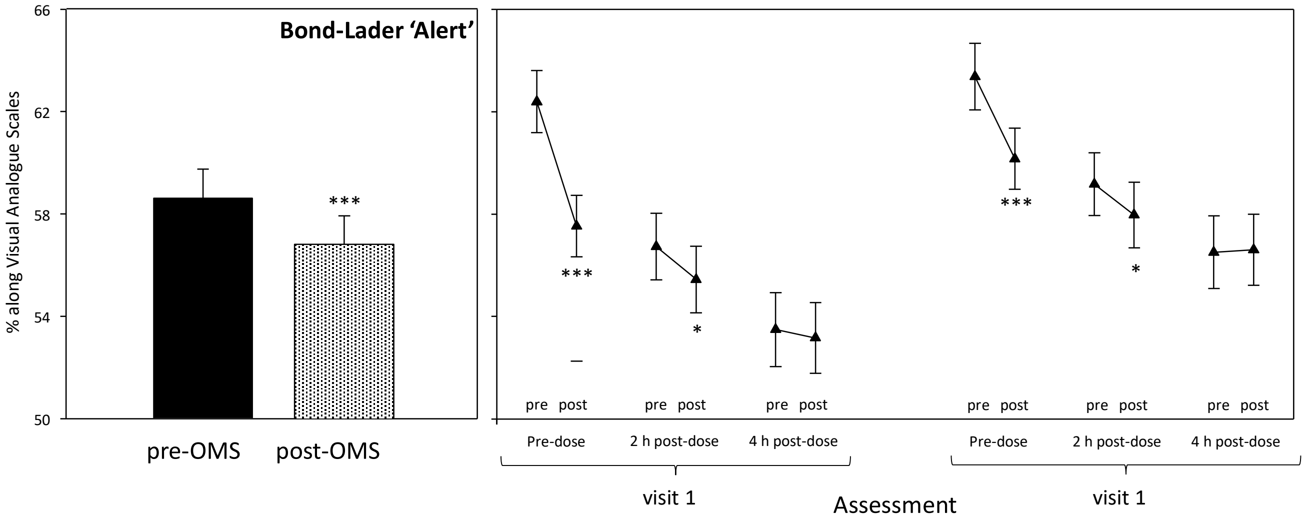

Figure 4. The effects of completing the OMS stressor on ratings of alertness (Bond-Lader Mood Scales). Left panel: the main effect of OMS completion averaged across all assessments [F (1, 246) = 30.84, p < 0.001]. Right panel: ratings of alertness before and after the OMS during each assessment on both visits. In this instance the interaction was between pre/post and assessment [F (2, 246) = 23.03, p < 0.001] with no visit ijteraction. Asterisks denote a significant difference between pre-OMS and post-OMS ratings during that assessment. *, p < 0.05; ***, p < 0.001.

Section II – Tables.

Table S1. Acute (Day 1) effects of green oat extract on Cognitive task performance………………… 5

Table S2. Chronic (Day 29) effects of green oat extract on Cognitive task performance 6

Table S3. Acute (Day 1) effects of green oat extract on Multitasking accuracy (subtractions and tracking). 7

Table S4. Acute (Day 1) effects of green oat extract on Multitasking speed (number of subtractions) and accuracy (tracking) 8

Table S5. Chronic (Day 29) effects of green oat extract on Multitasking accuracy (subtractions and tracking) 9

Table S6. Chronic (Day 29) effects of green oat extract on Multitasking speed (subtractions) and accuracy (tracking) 10

Table S7. Acute (Day 1) effects of green oat extract on the change in mood during the OMS........... 11

Table S8. Chronic (Day 29) effects of green oat extract on the change in mood during the OMS....... 13

Table S9. Profile of Mood States (POMS) sub-factor scores 14

Table S10. General Health Questionnaire-12 (GHQ-12) scores (PP population). 16

Table S11. Acute (Day 1) effects of green oat extract on galvanic skin response and heart data during the stressor 16

Table S12. Chronic (Day 29) effects of green oat extract on galvanic skin response and heart data 17

Table S13. Acute (Day 1) effects of green oat extract on salivary cortisol and α-amylase 18

Table S14. Chronic (Day 29) effects of green oat extract on salivary cortisol and α-amylase 19

nutrients

MDPI

Table 1. *Acute (Day 1) effects of green oat extract on Cognitive task performance*. Assessments comprised Corsi Blocks working memory task, Stroop task, Rapid Visual Information Processing task, and Numeric Working Memory task. Pre-dose baseline data are means (+sem); post-dose data (averaged and by assessment) are estimated means (+sem) from the ANCOVA analysis using the pre-dose baseline score and participant's age as covariates. F statistics (F) and probabilities (P) are from the ANCOVAs. treat = main effect of treatment; treat*Ass = treatment assessment interaction.

Day 1		Baseline (Day 1 pre-dose)		Post-Dose Averaged		Day 1 Post-Dose Assessment 2 hr		4 hr				
**Outcome**		**mean**	**sem**	**mean**	**sem**	**mean**	**sem**	**mean**	**sem**		**F**	**P**
**Corsi Blocks Working memory** (span) N = 132	Placebo	5.79	0.14	5.81	0.10	5.86	0.12	5.77	0.13	treat	3.66	0.014
	430 mg	5.98	0.14	5.99	0.10	5.99	0.12	5.98	0.13	treat x ass	0.20	0.898
	860 mg	6.00	0.14	5.65	0.10	5.66	0.12	5.64	0.13			
	1290 mg	5.73	0.15	6.10	0.11	6.07	0.13	6.14	0.13			
**Stroop Task** (% acc) N = 132	Placebo	98.92	0.33	97.98	0.29	97.83	0.35	98.13	0.35	treat	0.77	0.516
	430 mg	98.43	0.33	98.23	0.29	97.94	0.34	98.53	0.35	treat x ass	0.86	0.464
	860 mg	98.33	0.34	98.13	0.29	98.18	0.35	98.08	0.35			
	1290 mg	98.55	0.35	98.59	0.30	98.68	0.36	98.5	0.36			
**Stroop Task** (msec) N= 132	Placebo	823.7	25.5	775.4	11.5	770.8	11.7	779.9	14.1	treat	0.61	0.607
	430 mg	825.5	25.5	786.6	11.4	779.8	11.6	793.5	14.0	treat x ass	1.58	0.196
	860 mg	829.5	25.9	795.3	11.6	802.6	11.8	788.1	14.2			
	1290 mg	809.0	26.7	777.6	12.0	784.6	12.2	770.6	14.7			
**Rapid Visual Information Processing** (% acc) N=129	Placebo	57.35	3.54	62.90	1.68	63.53	1.95	62.27	1.80	treat	1.48	0.223
	430 mg	61.02	3.60	63.08	1.69	63.25	1.97	62.91	1.81	treat x ass	1.91	0.132
	860 mg	60.38	3.54	59.28	1.67	60.85	1.94	57.70	1.79			
	1290 mg	62.82	3.66	63.86	1.73	62.63	2.00	65.09	1.85			
**Rapid Visual Information Processing** (msec) N=129	Placebo	490.3	9.10	493.0	4.53	491.3	6.14	494.7	5.32	treat	1.58	0.197
	430 mg	497.6	9.24	493.2	4.58	486.1	6.22	500.2	5.38	treat x ass	0.55	0.65
	860 mg	493.3	9.10	505.2	4.51	502.9	6.12	507.4	5.30			
	1290 mg	490.0	9.39	497.5	4.65	496.1	6.31	499.0	5.54			
**Numeric Working Memory** (% acc) N=132	Placebo	95.69	0.70	96.86	0.55	96.72	0.58	97.00	0.69	treat	0.50	0.684
	430 mg	96.64	0.70	96.41	0.54	96.56	0.57	96.25	0.68	treat x ass	2.19	0.092
	860 mg	95.42	0.71	96.63	0.55	97.60	0.58	95.66	0.70			
	1290 mg	97.53	0.73	95.92	0.57	96.02	0.60	95.82	0.72			
**Numeric Working Memory** (msec) N=132	Placebo	925.1	43.1	916.5	17.7	926.5	20.2	906.5	19.0	treat	0.93	0.43
	430 mg	917.4	43.1	888.0	17.6	883.4	20.1	892.5	18.9	treat x ass	1.65	0.182
	860 mg	956.1	43.7	885.8	17.9	893.7	20.4	877.9	19.2			
	1290 mg	927.9	45.1	917.0	18.4	939.4	21.1	894.6	19.8			

Table 2. *Chronic (Day 29) effects of green oat extract on Cognitive task performance* in the PP population. Assessments comprised: Corsi Blocks working memory task, Stroop task, Rapid Visual Information Processing task, and Numeric Working Memory task. Pre-dose Visit 1 baseline data are means (+sem); post-dose Visit 2 data (averaged and by assessment) are estimated means (+sem) from the ANCOVA analysis using the pre-dose baseline score and participant's age as covariates. F statistics (F) and probabilities (P) are from the ANCOVA. treat = main effect of treatment; treat*Ass = treatment assessment interaction.

**Day 29**		**Baseline** (Day 1 pre-dose)		**Post-dose Averaged**		**Day 29 Assessment**								
						**Pre-dose**		**2 hr**		**4 hr**				
**Outcome**		**mean**	**sem**	**mean**	**sem**	**mean**	**sem**	**mean**	**sem**	**mean**	**sem**		**Fs**	**p**
**Corsi Blocks Working memory** (span) N=128	Placebo	5.83	0.14	5.75	0.09	5.58	0.12	5.80	0.13	5.88	0.12	treat	2.86	0.04
	430 mg	5.97	0.14	6.08	0.09	6.09	0.12	6.05	0.12	6.09	0.12	treat x ass	0.97	0.448
	860 mg	6.01	0.14	5.87	0.09	5.76	0.12	5.98	0.13	5.86	0.12			
	1290 mg	5.83	0.15	6.07	0.10	5.91	0.13	6.04	0.14	6.25	0.13			
**Stroop Task** (% acc) N=129	Placebo	99.04	0.34	98.38	0.23	98.30	0.31	98.05	0.36	98.79	0.31	treat	0.20	0.897
	430 mg	98.38	0.34	98.61	0.23	98.92	0.31	98.43	0.35	98.49	0.30	treat x ass	0.70	0.652
	860 mg	98.33	0.34	98.41	0.23	98.39	0.31	98.39	0.36	98.45	0.31			
	1290 mg	98.45	0.37	98.48	0.25	98.38	0.33	98.49	0.38	98.55	0.33			
**Stroop Task** (msec) N=129	Placebo	824.6	25.9	764.6	12.3	782.0	15.0	752.7	14.3	759.2	13.7	treat	0.44	0.726
	430 mg	824.2	25.9	777.7	12.2	794.9	14.9	780.4	14.2	757.8	13.7	treat x ass	1.43	0.203
	860 mg	827.7	26.3	782.7	12.4	817.7	15.2	761.8	14.4	768.5	13.9			
	1290 mg	802.6	28.1	768.6	13.3	796.8	16.2	764.3	15.4	744.7	14.9			
**Rapid Visual Information Processing** (% acc) N=125	Placebo	58.36	3.57	64.13	1.90	64.46	2.08	62.86	2.22	65.07	2.30	treat	0.53	0.662
	430 mg	61.37	3.63	63.64	1.95	63.76	2.14	64.47	2.28	62.71	2.36	treat x ass	2.20	0.044
	860 mg	61.02	3.57	61.58	1.89	65.18	2.07	62.03	2.21	57.54	2.29			
	1290 mg	64.38	3.82	64.87	2.03	65.93	2.22	66.06	2.36	62.61	2.45			
**Rapid Visual Information Processing** (msec) N=125	Placebo	486.6	9.18	492.4	4.25	485.6	4.99	492.2	5.97	499.4	5.5	treat	1.41	0.244
	430 mg	498.4	9.33	497.3	4.38	503.2	5.15	497.9	6.15	490.9	5.6	treat x ass	1.81	0.098
	860 mg	492.3	9.18	500.6	4.23	497.4	4.97	497.6	5.94	506.6	5.4			
	1290 mg	491.2	9.82	488.8	4.52	486.7	5.31	488.1	6.35	491.7	5.8			
**Numeric Working Memory** (% acc) N=129	Placebo	95.79	0.72	96.77	0.69	97.03	0.76	96.33	0.79	96.96	0.90	treat	0.78	0.51
	430 mg	96.53	0.72	96.16	0.68	96.87	0.76	96.46	0.79	95.16	0.89	treat x ass	0.69	0.658
	860 mg	95.52	0.73	96.31	0.70	96.96	0.77	96.33	0.80	95.63	0.91			
	1290 mg	97.74	0.78	95.23	0.75	95.72	0.83	95.00	0.87	94.97	0.98			
**Numeric Working Memory** (msec) N=129	Placebo	933.4	43.7	903.6	18.5	913.9	20.1	917.6	20.9	879.5	26.1	treat	1.69	0.174
	430 mg	920.5	43.7	878.1	18.5	886.0	20.0	872.8	20.9	875.3	26.0	treat x ass	1.38	0.224
	860 mg	958.7	44.3	895.7	18.8	896.4	20.3	882.9	21.2	907.7	26.4			
	1290 mg	923.5	47.4	938.2	20.1	973.7	21.7	931.1	22.6	909.9	28.2			

Table 3. *Acute (Day 1) effects of green oat extract on Multitasking accuracy (subtractions and tracking).* Assessments comprised 12 minutes of Serial Subtractions (3s, 7s, 17s) and the concomitant tracking task. Data for both tasks were converted to standardised Z scores for pooled analysis. Data is presented here averaged across tasks at each assessment (top of table) and averaged across assessments for each task (bottom of table). Pre-dose Visit 1 baseline data are means (+sem); post-dose data are estimated means (+sem) from the LMM analysis using the pre-dose baseline score and participant's age as covariates. F statistics (F) and probabilities (P) are from the LMM analysis. Treat = Main effect of treatment; Treat*Ass = treatment assessment interaction; Treat*Outcome = treatment outcome interaction.

**Day 1** N = 132		**Baseline** (Day 1 pre-dose)		**Post-dose Averaged**		**Day 1 Post-dose Assessment** 2 hr		4 hr			**Fs**	**p**
		**mean**	**sem**	**mean**	**sem**	**mean**	**sem**	**mean**	**sem**			
**Subtractions and Tracking Accuracy** Z scores — By Assessment	Placebo	-0.199	0.110	-0.253	0.106	-0.215	0.112	-0.291	0.112	Treat	1.53	0.209
	430 mg	-0.166	0.110	-0.078	0.106	-0.014	0.112	-0.143	0.112	Treat*Ass	0.38	0.767
	860 mg	0.020	0.111	0.035	0.107	0.128	0.114	-0.058	0.114			
	1290 mg	-0.087	0.115	0.020	0.111	0.076	0.117	-0.036	0.117			
		**Baseline** (visit 1 assessment 1) Subtractions		Tracking		**Post-dose Averaged** Subtractions		Tracking				
		**mean**	**sem**	**mean**	**sem**	**mean**	**sem**	**mean**	**sem**			
By Outcome	Placebo	-0.262	0.127	-0.135	0.126	-0.236	0.112	-0.270	0.112	Treat*Outcome	2.64	0.048
	430 mg	-0.160	0.126	-0.171	0.126	-0.070	0.112	-0.086	0.112			
	860 mg	0.135	0.128	-0.096	0.128	0.078	0.114	-0.008	0.114			
	1290 mg	-0.107	0.132	-0.067	0.132	-0.076	0.117	0.115	0.117			

Table 4. *Acute (Day 1) effects of green oat extract on Multitasking speed (number of subtractions) and accuracy (tracking).* Assessments comprised 12 minutes of Serial Subtractions (3s, 7s, 17s) and the concomitant tracking task. Data for both tasks were converted to standardised Z scores for pooled analysis. Data is presented here averaged across tasks at each assessment (top of table) and averaged across assessments for each task (bottom of table). Pre-dose Visit 1 baseline data are means (+sem); post-dose data are estimated means (+sem) from the LMM analysis using the pre-dose baseline score and participant's age as covariates. F statistics (F) and probabilities (P) are from the LMM analysis. Treat = Main effect of treatment; Treat*Ass = treatment assessment interaction; Treat*Outcome = treatment outcome interaction.

**Day 1**		**Baseline (Day 1 pre-dose)**		**Post-dose Averaged**		**Day 1 Post-dose Assessment** 2 hr		4 hr				
N = 132		**mean**	**sem**	**mean**	**sem**	**mean**	**sem**	**mean**	**sem**	**Fs**		**p**
**Subtractions Speed and Tracking Accuracy** Z scores	By Assessment: Placebo	-0.223	0.119	-0.200	0.099	-0.147	0.104	-0.253	0.104	Treat	1.11	0.349
	430 mg	-0.123	0.119	0.018	0.099	0.062	0.104	-0.026	0.104	Treat*Ass	0.23	0.875
	860 mg	-0.155	0.121	-0.003	0.100	0.027	0.105	-0.034	0.105			
	1290 mg	-0.162	0.125	0.011	0.104	0.029	0.109	-0.007	0.109			
		**Baseline** (visit 1 assessment 1) Subtractions		Tracking		**Post-dose Averaged** Subtractions		Tracking				
		**mean**	**sem**	**mean**	**sem**	**mean**	**sem**	**mean**	**sem**			
	By Outcome: Placebo	-0.312	0.130	-0.135	0.129	-0.130	0.104	-0.270	0.104	Treat*Outcome	8.26	0.000
	430 mg	-0.075	0.129	-0.171	0.129	0.123	0.104	-0.086	0.104			
	860 mg	-0.214	0.131	-0.096	0.131	0.002	0.105	-0.008	0.105			
	1290 mg	-0.257	0.135	-0.067	0.135	-0.093	0.109	0.115	0.109			

Table 5. *Chronic (Day 29) effects of green oat extract on Multitasking accuracy (subtractions and tracking)* in the PP population. Assessments comprised 12 minutes of Serial Subtractions (3s, 7s, 17s) and the concomitant tracking task. Data for both tasks were converted to standardised Z scores for pooled analysis. Data is presented here averaged across tasks at each assessment (top of table) and averaged across assessments for each task (bottom of table). Pre-dose Visit 1 baseline data are means (+sem); post-dose Visit 2 data are estimated means (+sem) from the LMM analysis using the pre-dose baseline score and participant's age as covariates. F statistics (F) and probabilities (P) are from the LMM analysis. Treat = Main effect of treatment; Treat*Ass = treatment assessment interaction; Treat*Outcome = treatment outcome interaction.

**Day 29**		**Baseline (Day 1 pre-dose)**		**Post-dose Averaged**		**Day 29 Assessment**								
						**Pre-dose**		**2 hr**		**4 hr**				
N = 126		**mean**	**sem**	**mean**	**sem**	**mean**	**sem**	**mean**	**sem**	**mean**	**sem**		**Fs**	**p**
**Subtractions and Tracking Accuracy** Z scores	By Assessment Placebo	-0.196	0.113	-0.085	0.101	-0.023	0.110	-0.040	0.110	-0.19	0.110	Treat	1.72	0.167
	430 mg	-0.189	0.113	0.125	0.103	0.184	0.111	0.136	0.111	0.055	0.111	Treat*Ass	0.27	0.952
	860 mg	0.030	0.115	0.113	0.000	0.138	0.112	0.175	0.112	0.027	0.112			
	1290 mg	-0.095	0.123	0.244	0.101	0.259	0.120	0.276	0.120	0.197	0.120			
		**Baseline** (visit 1 assessment 1)				**Post-dose Averaged**								
		Subtractions		Tracking		Subtractions		Tracking						
		**mean**	**sem**	**mean**	**sem**	**mean**	**sem**	**mean**	**sem**					
	By Outcome Placebo	-0.252	0.131	-0.141	0.130	0.011	0.106	-0.181	0.105			Treat*Outcome	10.48	0.000
	430 mg	-0.189	0.130	-0.189	0.130	0.107	0.106	0.143	0.106					
	860 mg	0.154	0.132	-0.095	0.132	0.178	0.108	0.048	0.108					
	1290 mg	-0.099	0.141	-0.092	0.141	0.099	0.115	0.389	0.115					

Table 6. *Chronic (Day 29) effects of green oat extract on Multitasking speed (subtractions) and accuracy (tracking)* in the PP population. Assessments comprised 15 minutes of Serial Subtractions (3s, 7s, 17s) and the concomitant tracking task. Data for both tasks was converted to standardised Z scores for pooled analysis. Data is presented here averaged across tasks at each assessment (top of table) and averaged across assessments for each task (bottom of table). Pre-dose Visit 1 baseline data are means (+sem); post-dose Visit 2 data are estimated means (+sem) from the LMM analysis using the pre-dose baseline score and participant's age as covariates. F statistics (F) and probabilities (P) are from the LMM analysis. Treat = Main effect of treatment; Treat*Ass = treatment assessment interaction; Treat*Outcome = treatment outcome interaction.

**Day 29** **N = 126**			**Baseline (Day 1 pre-dose)**		**Post-dose Averaged**		**Day 29 Assessment** **Pre-dose**		**2 hr**		**4 hr**				
			**mean**	**sem**	**mean**	**sem**	**mean**	**sem**	**mean**	**sem**	**mean**	**sem**		**Fs**	**p**
**Subtractions Speed and Tracking Accuracy** Z scores	By Assessment	Placebo	-0.211	0.123	-0.096	0.103	-0.052	0.110	-0.047	0.110	-0.18	0.110	Treat	1.64	0.183
		430 mg	-0.130	0.123	0.185	0.104	0.229	0.111	0.224	0.111	0.102	0.111	Treat*Ass	0.36	0.903
		860 mg	-0.146	0.125	0.102	0.105	0.091	0.113	0.166	0.113	0.050	0.113			
		1290 mg	-0.176	0.134	0.192	0.113	0.193	0.120	0.226	0.120	0.158	0.120			
			**Baseline** (visit 1 assessment 1) Subtractions		Tracking		**Post-dose Averaged** Subtractions		Tracking						
			**mean**	**sem**	**mean**	**sem**	**mean**	**sem**	**mean**	**sem**					
	By Outcome	Placebo	-0.282	0.134	-0.141	0.134	-0.001	0.107	-0.191	0.107			Treat*Outcome	20.41	0.000
		430 mg	-0.070	0.134	-0.189	0.134	0.226	0.107	0.143	0.107					
		860 mg	-0.198	0.136	-0.095	0.136	0.156	0.109	0.048	0.109					
		1290 mg	-0.260	0.145	-0.092	0.145	-0.004	0.117	0.389	0.117					

Table 7. *Acute (Day 1) effects of green oat extract on the change in mood during the OMS*. Mood was measured before (pre) and after (post) the stressor. Assessments comprised the State-Trait Anxiety Inventory and Bond-Lader Mood Scales. Pre-dose baseline data are means (+sem); post-dose data are estimated means (+sem) from the LMM analysis using the pre-dose baseline score and participant's age as covariates. F statistics (F) and probabilities (P) are from the LMM analysis. Treat = Main effect of treatment; Treat x Ass = treatment assessment interaction.

Day 1			Baseline (Day 1 pre-dose)		Post-dose Averaged		Day 1 Post-dose Assessment 2 hr		4 hr				
**Outcome**			**mean**	sem	**mean**	sem	**mean**	sem	**mean**	sem		F	P
**State Trait Anxiety Inventory** N = 131	placebo	pre	38.56	1.78	41.62	1.77	41.24	1.85	42.00	1.85	Treat	1.08	0.362
		post	45.94	1.78	43.77	1.77	44.00	1.85	43.53	1.85	Treat x ass	0.31	0.82
	430 mg	pre	36.27	1.80	36.94	1.80	36.36	1.87	37.52	1.87			
		post	43.48	1.80	39.92	1.80	40.36	1.87	39.48	1.87			
	860 mg	pre	37.00	1.80	38.62	1.80	38.94	1.87	38.29	1.87			
		post	45.67	1.80	41.65	1.80	42.03	1.87	41.27	1.87			
	1290 mg	pre	36.74	1.86	38.23	1.86	38.26	1.93	38.19	1.93			
		post	43.90	1.86	40.76	1.86	41.08	1.93	40.45	1.93			
**Bond-Lader Alert** N = 130	placebo	pre	61.19	2.41	51.55	2.51	53.75	2.61	49.34	2.61	Treat	1.69	0.173
		post	56.07	2.41	51.61	2.51	52.99	2.61	50.22	2.61	Treat x ass	0.80	0.496
	430 mg	pre	65.05	2.45	59.13	2.55	61.01	2.65	57.26	2.65			
		post	59.56	2.45	58.28	2.55	58.98	2.65	57.57	2.65			
	860 mg	pre	60.89	2.45	53.60	2.55	54.15	2.65	53.05	2.65			
		post	56.02	2.45	52.43	2.55	53.32	2.65	51.54	2.65			
	1290 mg	pre	63.64	2.56	56.62	2.68	58.13	2.78	55.10	2.78			
		post	60.51	2.56	56.32	2.68	57.72	2.78	54.93	2.78			
**Bond-Lader Content** N = 130	placebo	pre	64.85	2.44	59.91	2.39	60.62	2.48	59.20	2.48	Treat	1.08	0.362
		post	58.31	2.44	59.13	2.39	59.35	2.48	58.91	2.48	Treat x ass	1.25	0.293
	430 mg	pre	68.93	2.47	65.88	2.43	66.72	2.51	65.04	2.51			
		post	61.60	2.47	63.59	2.43	63.91	2.51	63.27	2.51			
	860 mg	pre	66.39	2.47	63.56	2.43	63.09	2.51	64.02	2.51			
		post	59.80	2.47	62.03	2.43	61.38	2.51	62.68	2.51			
	1290 mg	pre	68.08	2.59	65.22	2.55	65.05	2.64	65.39	2.64			
		post	64.29	2.59	64.33	2.55	64.92	2.64	63.75	2.64			
**Bond-Lader Calm** N = 130	placebo	pre	58.66	2.86	54.52	2.50	54.68	2.65	54.35	2.65	Treat	0.38	0.769
		post	51.54	2.86	51.15	2.50	51.15	2.65	51.16	2.65	Treat x ass	0.88	0.45

	430 mg	pre	59.24	2.90	56.56	2.54	56.02	2.69	57.11	2.69
		post	47.23	2.90	54.94	2.54	54.59	2.69	55.29	2.69
	860 mg	pre	57.44	2.90	56.62	2.54	56.06	2.69	57.18	2.69
		post	49.67	2.90	55.45	2.54	53.73	2.69	57.17	2.69
	1290 mg	pre	59.55	3.04	55.26	2.66	55.33	2.82	55.18	2.82
		post	50.73	3.04	52.84	2.66	53.05	2.82	52.63	2.82

Table 8. *Chronic (Day 29) effects of green oat extract on the change in mood during the OMS* (PP population). Mood was measured before (pre) and after (post) the stressor. Assessments comprised the State-Trait Anxiety Inventory and Bond-Lader Mood Scales. Pre-dose baseline data are means (+sem); post-dose data are estimated means (+sem) from the LMM analysis using the pre-dose baseline score and participant's age as covariates. F statistics (F) and probabilities (P) are from the LMM analysis. Treat = Main effect of treatment; Treat x Ass = treatment assessment interaction.

**Day 29**			**Baseline (Day 1 pre-dose)**		**Post-dose Averaged**		**Day 29 Assessment**							F	P
							**Pre-dose**		**2 hr**		**4 hr**				
**Outcome**			**mean**	sem	**mean**	sem	**mean**	sem	**mean**	sem	**mean**	sem			
**State Trait Anxiety Inventory** N = 125	placebo	pre	38.52	1.83	39.18	1.71	39.35	1.80	39.21	1.80	38.97	1.80	Treat	0.75	0.524
		post	46.03	1.83	41.72	1.71	42.27	1.80	41.58	1.80	41.30	1.80	Treat x ass	1.39	0.216
	430 mg	pre	35.97	1.86	36.93	1.73	35.84	1.83	37.66	1.83	37.28	1.83			
		post	43.30	1.86	40.54	1.73	41.38	1.83	42.25	1.83	37.98	1.83			
	860 mg	pre	36.94	1.86	35.24	1.73	34.29	1.83	35.72	1.83	35.72	1.83			
		post	45.50	1.86	38.80	1.73	38.53	1.83	39.25	1.83	38.62	1.83			
	1290 mg	pre	36.93	1.99	36.04	1.85	35.39	1.96	36.25	1.96	36.46	1.96			
		post	44.43	1.99	39.56	1.85	39.96	1.96	40.18	1.96	38.54	1.96			
**Bond-Lader Alert** N = 124	placebo	pre	61.43	2.45	56.76	2.39	60.64	2.56	57.27	2.55	52.37	2.55	Treat	1.62	0.189
		post	56.03	2.45	55.21	2.39	56.85	2.56	55.64	2.55	53.14	2.55	Treat x ass	1.93	0.074
	430 mg	pre	66.04	2.49	62.56	2.42	65.79	2.59	62.33	2.59	59.56	2.59			
		post	60.37	2.49	61.39	2.42	61.24	2.59	61.44	2.59	61.48	2.59			
	860 mg	pre	61.01	2.49	59.00	2.42	63.55	2.59	58.64	2.59	54.81	2.59			
		post	55.95	2.49	57.24	2.42	60.35	2.59	56.45	2.59	54.94	2.59			
	1290 mg	pre	63.53	2.71	62.81	2.64	66.41	2.82	60.92	2.82	61.12	2.82			
		post	60.57	2.71	61.85	2.64	65.03	2.82	60.84	2.82	59.66	2.82			
**Bond-Lader Content** N = 124	placebo	pre	65.04	2.47	63.81	2.29	64.30	2.40	64.42	2.39	62.72	2.39	Treat	0.87	0.461
		post	58.41	2.47	61.57	2.29	60.56	2.40	61.70	2.39	62.45	2.39	Treat x ass	1.04	0.396
	430 mg	pre	69.56	2.51	68.22	2.33	69.34	2.43	68.03	2.43	67.29	2.43			
		post	62.28	2.51	65.82	2.33	64.36	2.43	65.52	2.43	67.59	2.43			
	860 mg	pre	66.57	2.51	67.79	2.33	69.44	2.43	67.10	2.43	66.81	2.43			
		post	59.64	2.51	65.99	2.33	65.85	2.43	65.66	2.43	66.47	2.43			
	1290 mg	pre	67.25	2.73	67.67	2.53	68.93	2.64	66.92	2.64	67.17	2.64			
		post	62.78	2.73	66.20	2.53	66.88	2.64	64.96	2.64	66.74	2.64			
**Bond-Lader Calm** N = 124	placebo	pre	58.97	2.92	56.18	2.48	56.13	2.67	56.67	2.66	55.74	2.66	Treat	0.43	0.734
		post	51.67	2.92	54.02	2.48	54.91	2.67	52.65	2.66	54.49	2.66	Treat x ass	1.20	0.306
	430 mg	pre	59.69	2.97	58.10	2.52	58.59	2.71	58.08	2.71	57.64	2.71			
		post	47.45	2.97	56.36	2.52	53.91	2.71	56.14	2.71	59.03	2.71			
	860 mg	pre	57.44	2.97	59.92	2.52	62.34	2.71	58.30	2.71	59.13	2.71			
		post	49.58	2.97	56.43	2.52	56.30	2.71	55.08	2.71	57.91	2.71			
	1290 mg	pre	59.91	3.23	59.72	2.74	61.26	2.95	58.46	2.95	59.44	2.95			
		post	50.80	3.23	57.96	2.74	59.13	2.95	56.39	2.95	58.37	2.95			

nutrients

MDPI

Table 9. Profile of Mood States (POMS) sub-factor scores (PP population). Data are raw means (+sem) pre and post-dose on the first (Day 1 – i.e. acute) and last (Day 29 – i.e. chronic) day of supplementation. F statistics (F) and probabilities (P) are from a two-factor [treatment x pre/post dose) ANOVA.

**N = 125**		**Day 1**				**Day 29**						
		**Pre-dose**		**Post-dose**		**Pre-dose**		**Post-dose**				
**Outcome**		**mean**	**sem**	**mean**	**sem**	**mean**	**sem**	**mean**			**Fs**	**p**
**Tension/Anxiety**	Placebo	10.24	1.09	11.15	1.20	9.58	1.07	8.36	1.06	Treat*vis	0.14	0.935
	430 mg	7.12	1.09	8.21	1.20	6.00	1.07	6.52	1.06	Treat*vis*ass	0.79	0.501
	860 mg	9.13	1.13	9.23	1.24	7.52	1.10	7.65	1.09			
	1290 mg	9.07	1.19	9.89	1.30	7.54	1.16	7.32	1.15			
**Friendliness**	Placebo	15.00	0.66	14.79	0.73	15.06	0.65	15.91	0.70	Treat*vis	0.40	0.757
	430 mg	16.97	0.66	16.18	0.73	17.15	0.65	16.39	0.70	Treat*vis*ass	1.60	0.194
	860 mg	15.90	0.68	16.45	0.76	16.55	0.68	16.52	0.72			
	1290 mg	16.07	0.72	14.43	0.80	16.46	0.71	15.68	0.76			
**Fatigue/Inertia**	Placebo	7.82	0.95	10.24	0.99	6.55	0.80	7.06	0.93	Treat*vis	0.63	0.597
	430 mg	5.73	0.95	6.30	0.99	4.42	0.80	5.33	0.93	Treat*vis*ass	1.63	0.186
	860 mg	7.10	0.98	8.65	1.03	6.16	0.82	7.29	0.96			
	1290 mg	6.36	1.03	7.61	1.08	5.07	0.86	5.68	1.01			
**Depression/Dejection**	Placebo	4.67	1.03	6.30	1.16	4.67	1.07	4.12	1.02	Treat*vis	0.33	0.804
	430 mg	4.09	1.03	4.55	1.16	3.09	1.07	3.12	1.02	Treat*vis*ass	1.05	0.372
	860 mg	4.87	1.06	5.55	1.20	3.84	1.10	3.26	1.05			
	1290 mg	5.29	1.11	5.82	1.26	4.96	1.16	5.04	1.11			
**Confusion/Bewilderment**	Placebo	10.18	0.92	11.06	1.12	9.91	0.99	9.88	1.02	Treat*vis	2.35	0.076
	430 mg	8.03	0.92	9.33	1.12	7.33	0.99	8.27	1.02	Treat*vis*ass	1.15	0.330
	860 mg	10.00	0.95	11.97	1.15	8.48	1.02	8.16	1.05			
	1290 mg	8.21	1.00	10.21	1.21	8.07	1.08	8.50	1.11			
**Anger/Hostility**	Placebo	3.97	0.78	4.91	0.89	4.03	0.87	4.09	0.80	Treat*vis	0.11	0.952
	430 mg	4.15	0.78	3.24	0.89	3.18	0.87	3.06	0.80	Treat*vis*ass	1.11	0.349
	860 mg	4.74	0.81	4.68	0.92	4.77	0.90	4.23	0.82			
	1290 mg	4.96	0.85	4.89	0.97	4.25	0.95	4.07	0.87			
**Vigour/Activity**	Placebo	16.36	1.26	15.67	1.35	15.49	1.12	15.91	1.26	Treat*vis	2.31	0.080
	430 mg	19.06	1.26	17.85	1.35	20.39	1.12	19.03	1.26	Treat*vis*ass	0.58	0.629
	860 mg	17.42	1.30	16.23	1.39	18.29	1.15	18.71	1.30			
	1290 mg	18.25	1.36	16.04	1.47	20.11	1.21	19.50	1.36			
**Total Mood Disturbance**	Placebo	20.52	4.61	28.00	5.03	19.24	4.71	17.61	4.77	Treat*vis	0.26	0.856

	430 mg	10.06	4.61	13.79	5.03	3.64	4.71	7.27	4.77	Treat*vis*ass	1.38	0.252
	860 mg	18.42	4.75	23.84	5.19	12.48	4.86	11.88	4.92			
	1290 mg	15.64	5.00	22.39	5.46	9.79	5.12	11.11	5.18			

Table 10. *General Health Questionnaire-12 (GHQ-12) scores (PP population).* Data are raw means (+sem) pre and post-dose on the first (Day 1 – i.e. acute) and last (Day 29 – i.e. chronic) day of supplementation. F statistics (F) and probabilities (P) are from a two-factor [treatment x pre/post dose) ANOVA.

		Day 1				Day 29						
		Pre-dose		Post-dose		Pre-dose		Post-dose				
**Outcome**		**mean**	**sem**	**mean**	**sem**	**mean**	**sem**	**mean**			**Fs**	**p**
**General Health Questionnaire – 12** N = 125	Placebo	10.73	0.56	10.24	0.63	8.79	0.58	8.70	0.65	Treat*vis	0.02	0.997
	430 mg	9.55	0.56	9.36	0.63	7.85	0.58	7.85	0.65	Treat*vis*ass	0.96	0.414
	860 mg	10.19	0.57	9.91	0.64	8.50	0.59	8.50	0.66			
	1290 mg	10.54	0.61	10.61	0.68	9.25	0.63	8.61	0.70			

Table 11. *Acute (Day 1) effects of green oat extract on galvanic skin response and heart data during the stressor*. Pre-dose Day 1 baseline data are means (+sem); post-dose data are estimated means (+sem) from the LMM analysis using the pre-dose Visit 1 baseline score and participant's age as covariates. F statistics (F) and probabilities (P) are from the LMM analysis. Treat = Main effect of treatment; Treat*Ass = treatment assessment interaction.

**Day 1**		**Baseline (Day 1 pre-dose)**		**Post-dose Averaged**		**Day 1 Post-dose Assessment**						
						**2 hr**		**4 hr**				
**Outcome**		**mean**	**sem**	**mean**	**sem**	**mean**	**sem**	**mean**	**sem**		**Fs**	**p**
**Galvanic Skin Response** (microsiemens) N = 132	Placebo	3.94	0.90	2.72	0.69	3.10	0.73	2.35	0.73	Treat	1.31	0.275
	430 mg	3.62	0.90	3.19	0.69	3.38	0.73	3.01	0.73	Treat*Ass	1.07	0.360
	860 mg	4.48	0.92	2.01	0.70	2.47	0.74	1.54	0.74			
	1290 mg	2.42	0.95	1.34	0.73	1.28	0.76	1.41	0.76			
**Heart Rate** (Beats per Minute) N = 132	Placebo	-1.14	1.65	-3.56	1.69	-3.10	1.73	-4.03	1.72	Treat	1.29	0.282
	430 mg	1.14	1.65	-0.41	1.69	0.19	1.72	-1.01	1.73	Treat*Ass	4.47	0.004
	860 mg	0.53	1.67	1.06	1.72	0.94	1.75	1.18	1.75			
	1290 mg	-0.78	1.73	-1.04	1.77	-2.07	1.81	-0.01	1.81			

Table 12. *Chronic (Day 29) effects of green oat extract on galvanic skin response and heart data (PP population).* Pre-dose Day 1 baseline data are means (+sem); Day 29 data are estimated means (+sem) from the LMM analysis using the pre-dose Day 1 baseline score and participant's age as covariates. F statistics (F) and probabilities (P) are from the LMM analysis. Treat = Main effect of treatment; Treat*Ass = treatment assessment interaction.

**Day 29**		**Baseline (Day 1 pre-dose)**		**Post-dose Averaged**		**Day 29 Post-dose Assessment 1**		**2**		**3**				
**Outcome**		**mean**	**sem**	**mean**	**sem**	**mean**	**sem**	**mean**	**sem**	**mean**	**sem**		**F**	**p**
**Galvanic Skin Response** (microsiemens) N = 126	Placebo	4.03	0.92	2.20	0.45	2.44	0.47	2.09	0.47	2.08	0.47	Treat	1.50	0.217
	430 mg	3.43	0.92	1.66	0.45	1.92	0.47	1.50	0.47	1.55	0.47	Treat*Ass	3.40	0.003
	860 mg	4.57	0.93	1.81	0.46	2.43	0.47	1.32	0.47	1.67	0.47			
	1290 mg	2.51	1.00	0.81	0.49	1.65	0.51	0.36	0.51	0.42	0.51			
**Heart Rate** (Beats per Minute) N = 126	Placebo	-1.22	1.70	-1.72	1.71	-0.85	1.79	-1.24	1.79	-3.08	1.79	Treat	0.22	0.884
	430 mg	1.10	1.70	0.01	1.71	-0.08	1.79	0.01	1.79	0.10	1.79	Treat*Ass	2.11	0.05
	860 mg	0.47	1.73	-0.24	1.74	-1.20	1.81	0.52	1.81	-0.03	1.81			
	1290 mg	-0.28	1.85	-0.16	1.86	0.81	1.94	-0.87	1.94	-0.42	1.94			

Table 13. *Acute (Day 1) effects of green oat extract on salivary cortisol and α-amylase.* Pre-dose Day 1 baseline data are means (+sem); post-dose data are estimated means (+sem) from the LMM analysis using the pre-dose Day 1 baseline score and participant's age as covariates. F statistics (F) and probabilities (P) are from the LMM analysis. Treat = Main effect of treatment; Treat*Ass = treatment assessment interaction.

**Day 1**			**Baseline (Day 1 pre-dose)**		**Post-dose Averaged**		**Day 1 Post-dose Assessment**						
							**2 hr**		**4 hr**				
**Outcome**			**mean**	**sem**	**mean**	**sem**	**mean**	**sem**	**mean**	**sem**		**F**	**P**
**Cortisol** N = 128	placebo	pre	0.343	0.041	0.169	0.023	0.146	0.028	0.191	0.028	Treat	0.891	0.448
		post	0.341	0.041	0.203	0.024	0.167	0.028	0.24	0.028	Treat*prepost	0.18	0.91
	430 mg	pre	0.281	0.041	0.145	0.023	0.137	0.028	0.152	0.028	Treat*pp*ass	0.465	0.707
		post	0.297	0.041	0.178	0.023	0.166	0.028	0.19	0.028			
	860 mg	pre	0.251	0.042	0.198	0.024	0.18	0.029	0.216	0.029			
		post	0.274	0.042	0.212	0.024	0.216	0.029	0.209	0.029			
	1290 mg	pre	0.25	0.043	0.153	0.024	0.131	0.029	0.175	0.029			
		post	0.311	0.043	0.178	0.024	0.161	0.029	0.195	0.029			
**α-amylase** N = 127	placebo	pre	182.3	25.7	217.5	23.1	188.5	25.8	246.5	25.7	Treat	0.436	0.727
		post	221.1	26.4	197.7	23.5	169.5	26.2	226.0	26.2	Treat*prepost	1.19	0.313
	430 mg	pre	176.3	26.1	195.0	23.4	200.0	25.9	190.1	26.1	Treat*pp*ass	1.005	0.391
		post	215.8	26.1	214.1	23.5	196.5	25.9	231.7	26.3			
	860 mg	pre	178.3	26.5	186.0	23.9	181.3	26.6	190.8	26.5			
		post	181.7	26.7	172.6	24.1	150.8	26.8	194.4	26.8			
	1290 mg	pre	175.5	26.9	219.6	24.1	226.0	26.7	213.3	26.7			
		post	209.0	26.9	205.6	24.2	224.9	26.7	186.2	26.9			

Table 14. *Chronic (Day 29) effects of green oat extract on salivary cortisol and α-amylase (PP population)*. Pre-dose Day 1 baseline data are means (+sem); Day 29 data are estimated means (+sem) from the LMM analysis using the pre-dose Day 1 baseline score and participant's age as covariates. F statistics (F) and probabilities (P) are from the LMM analysis. Treat = Main effect of treatment; Treat*Ass = treatment assessment interaction.

**Day 29**			**Baseline** (Day 1 pre-dose)		**Post-dose Averaged**		**Day 29 Post-dose Assessment** **1**		**2**		**3**			F	P
**Outcome**			**mean**	sem	**mean**	sem	**mean**	sem	**mean**	sem	**mean**	sem			
**Cortisol** N = 125	placebo	pre	0.35	0.042	0.193	0.017	0.257	0.022	0.152	0.022	0.169	0.022	Treat	1.05	0.373
		post	0.343	0.042	0.194	0.017	0.234	0.023	0.144	0.023	0.203	0.023	Treat*prepost	2.103	0.099
	430 mg	pre	0.283	0.042	0.198	0.017	0.268	0.022	0.154	0.022	0.171	0.022	Treat*pp*ass	0.658	0.684
		post	0.301	0.042	0.235	0.017	0.296	0.022	0.191	0.022	0.218	0.022			
	860 mg	pre	0.25	0.043	0.182	0.018	0.268	0.023	0.119	0.023	0.158	0.023			
		post	0.279	0.043	0.178	0.018	0.217	0.023	0.135	0.023	0.181	0.023			
	1290 mg	pre	0.254	0.045	0.212	0.018	0.301	0.024	0.16	0.024	0.177	0.024			
		post	0.315	0.045	0.203	0.018	0.291	0.024	0.156	0.024	0.16	0.024			
**α-amylase** N = 124	placebo	pre	184.5	25.7	173.2	19.9	157.2	22.8	156.7	22.9	205.7	22.8	Treat	0.753	0.523
		post	225.4	26.5	178.1	20.1	178.6	23.3	154.7	23.3	200.9	23.3	Treat*prepost	1.088	0.353
	430 mg	pre	171.0	26.1	187.0	20.2	185.5	23.1	181.3	23.1	194.3	23.1	Treat*pp*ass	0.116	0.995
		post	208.5	26.1	177.5	20.2	179.0	23.1	173.0	23.1	180.6	23.3			
	860 mg	pre	179.2	26.5	144.8	20.5	121.4	23.7	148.2	23.5	164.9	23.5			
		post	181.0	26.8	148.4	20.6	140.2	23.7	140.2	23.7	164.7	23.7			
	1290 mg	pre	170.7	27.9	193.6	21.5	169.7	24.7	195.4	24.7	215.7	24.7			
		post	207.8	27.9	172.0	21.5	166.5	24.7	162.3	24.7	187.2	24.7			

1. Kirschbaum, C.; Pirke, K.-M.; Hellhammer, D.H. The ‘trier social stress test’–a tool for investigating psychobiological stress responses in a laboratory setting. *Neuropsychobiology* **1993**, *28*, 76-81.
2. Scholey, A.; Haskell, C.; Robertson, B.; Kennedy, D.; Milne, A.; Wetherell, M. Chewing gum alleviates negative mood and reduces cortisol during acute laboratory psychological stress. *Physiology and Behavior* **2009**, *97*, 304-312.
3. Kennedy, D.O.; Little, W.; Haskell, C.F.; Scholey, A.B. Anxiolytic effects of a combination of melissa officinalis and valeriana officinalis during laboratory induced stress. *Phytotherapy Research* **2006**, *20*, 96-102.
4. Kennedy, D.O.; Little, W.; Scholey, A.B. Attenuation of laboratory-induced stress in humans after acute administration of melissa officinalis (lemon balm). *Psychosomatic Medicine* **2004**, *66*, 607-613.
5. Haskell, C.F.; Robertson, B.; Jones, E.; Forster, J.; Jones, R.; Wilde, A.; Maggini, S.; Kennedy, D.O. Effects of a multi-vitamin/mineral supplement on cognitive function and fatigue during extended multi-tasking. *Hum Psychopharmacol* **2010**, *25*, 448-461.
